# Supplementary material for: IsomiR-eQTL: A Cancer-Specific Expression Quantitative Trait Loci Database of miRNAs and Their Isoforms
Source: Int J Mol Sci. 2022 Oct 18;23(20):12493. doi: 10.3390/ijms232012493 (PMC9604134; doi:10.3390/ijms232012493)
Supplement: Supplementary file 1 [file ijms-23-12493-s001.zip › ijms-1910998-Supplementary.pptx]

## Slide 1
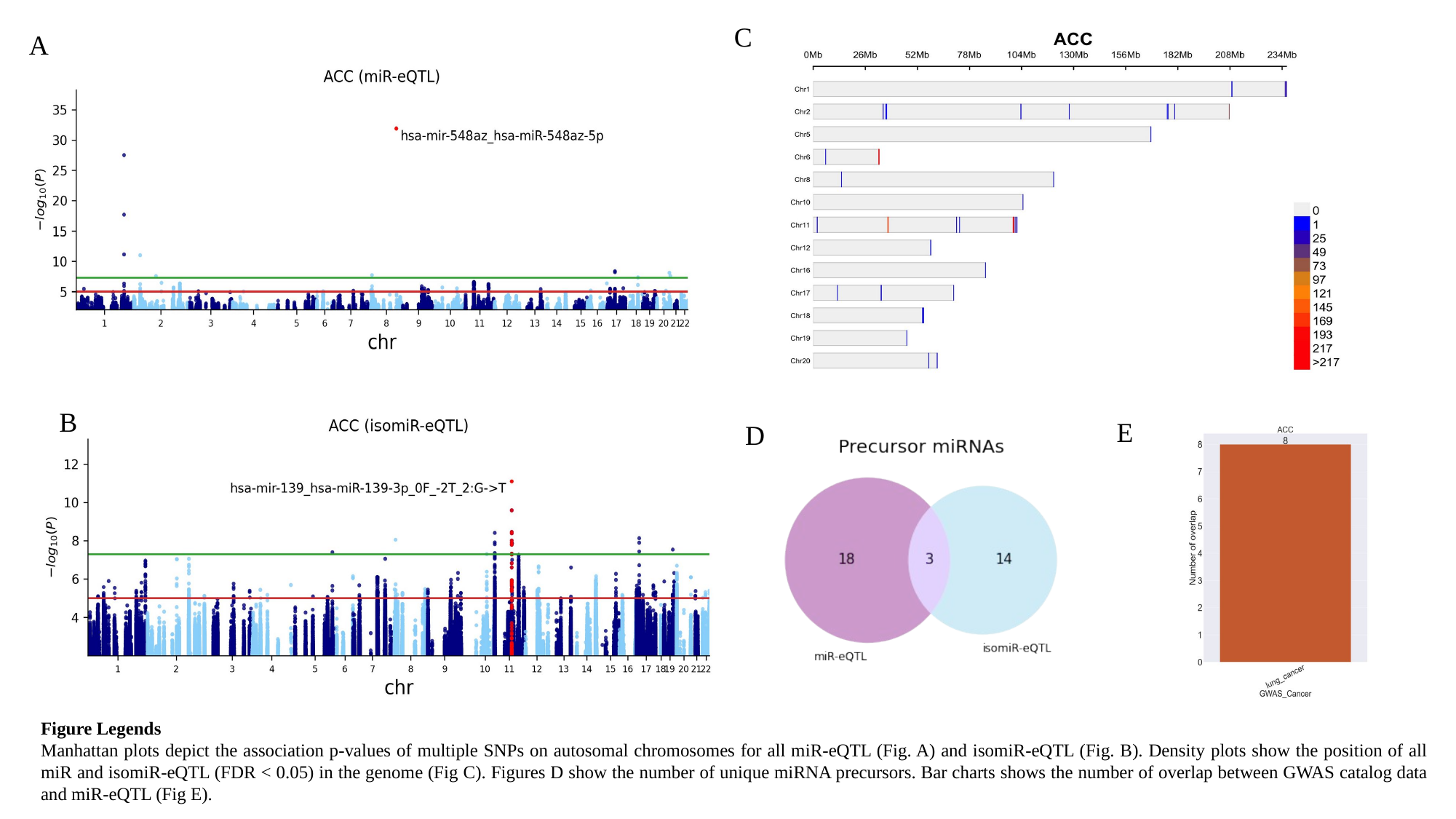

C
A
B
E
D
Figure Legends
Manhattan plots depict the association p-values of multiple SNPs on autosomal chromosomes for all miR-eQTL (Fig. A) and isomiR-eQTL (Fig. B). Density plots show the position of all miR and isomiR-eQTL (FDR < 0.05) in the genome (Fig C). Figures D show the number of unique miRNA precursors. Bar charts shows the number of overlap between GWAS catalog data and miR-eQTL (Fig E).

## Slide 2
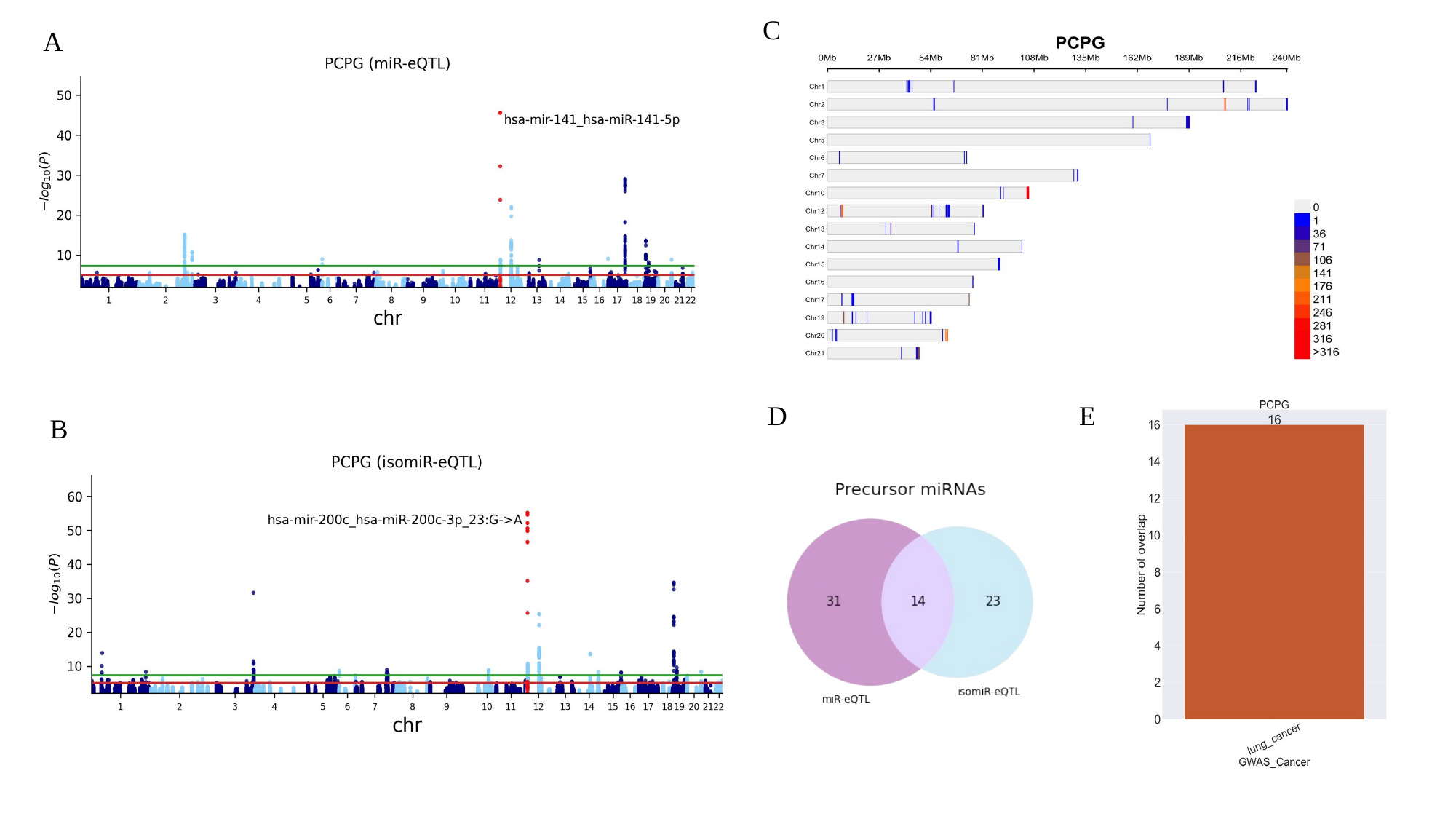

C
A
E
D
B

## Slide 3
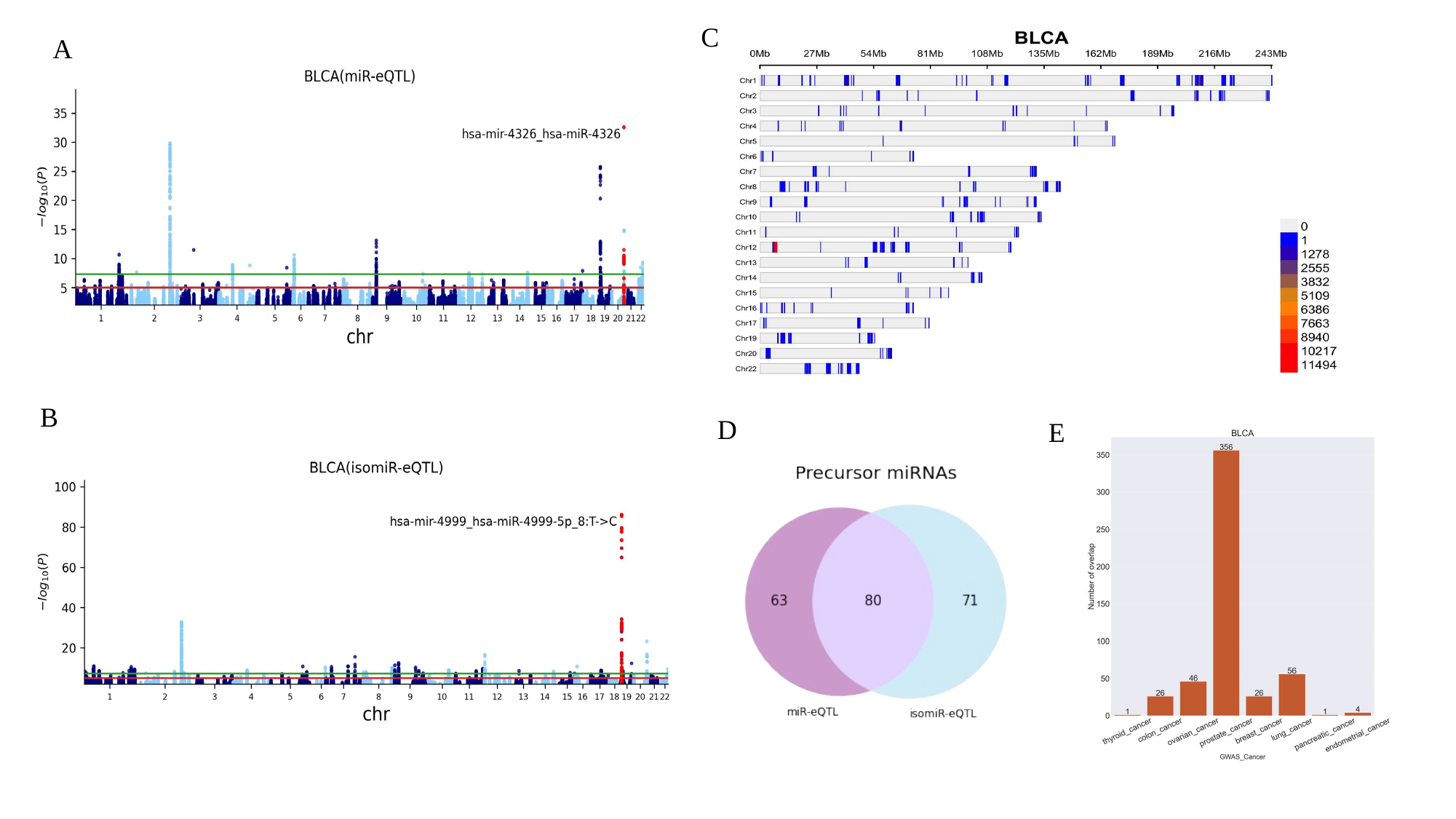

C
A
B
D
E

## Slide 4
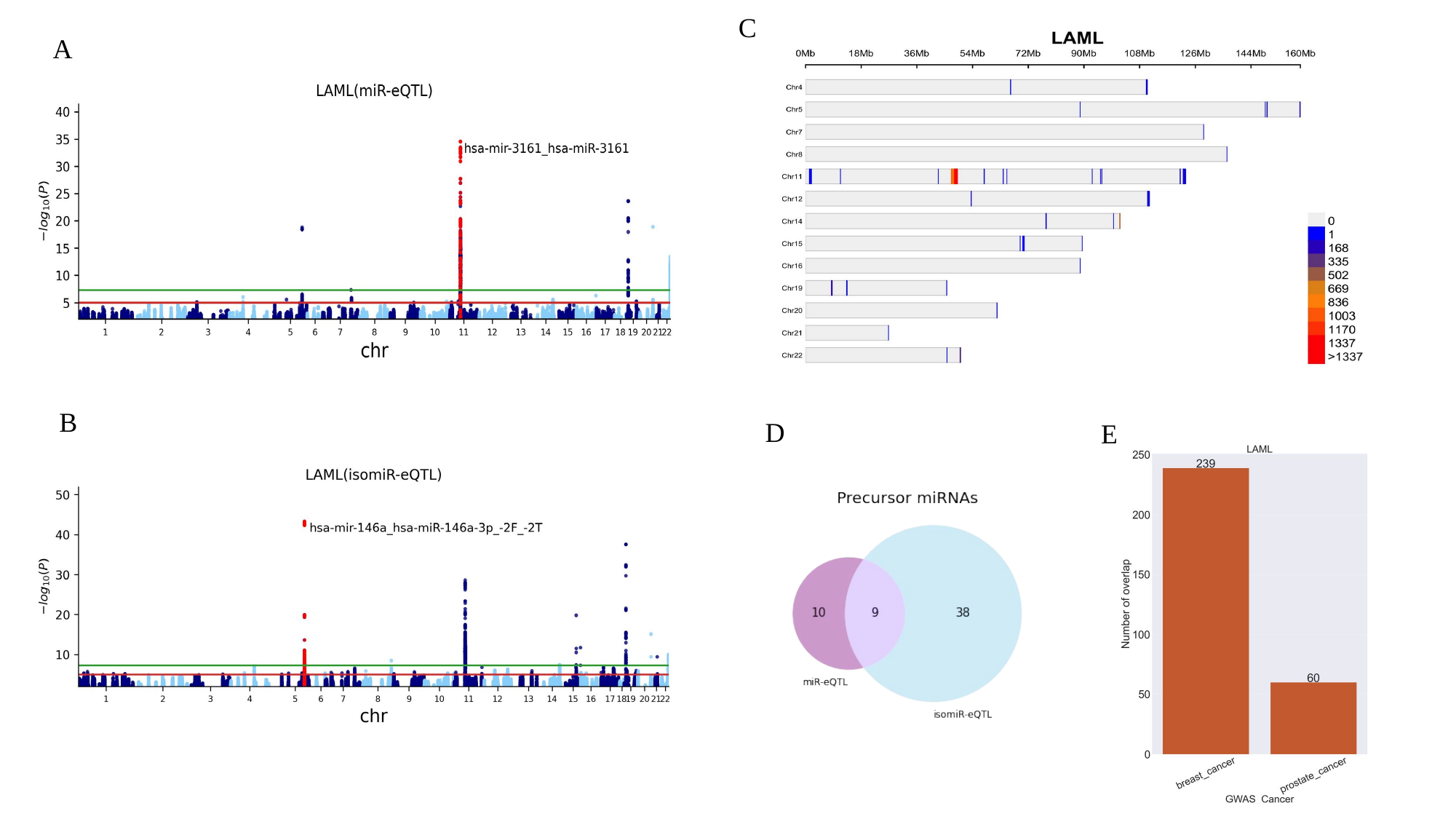

C
A
B
D
E

## Slide 5
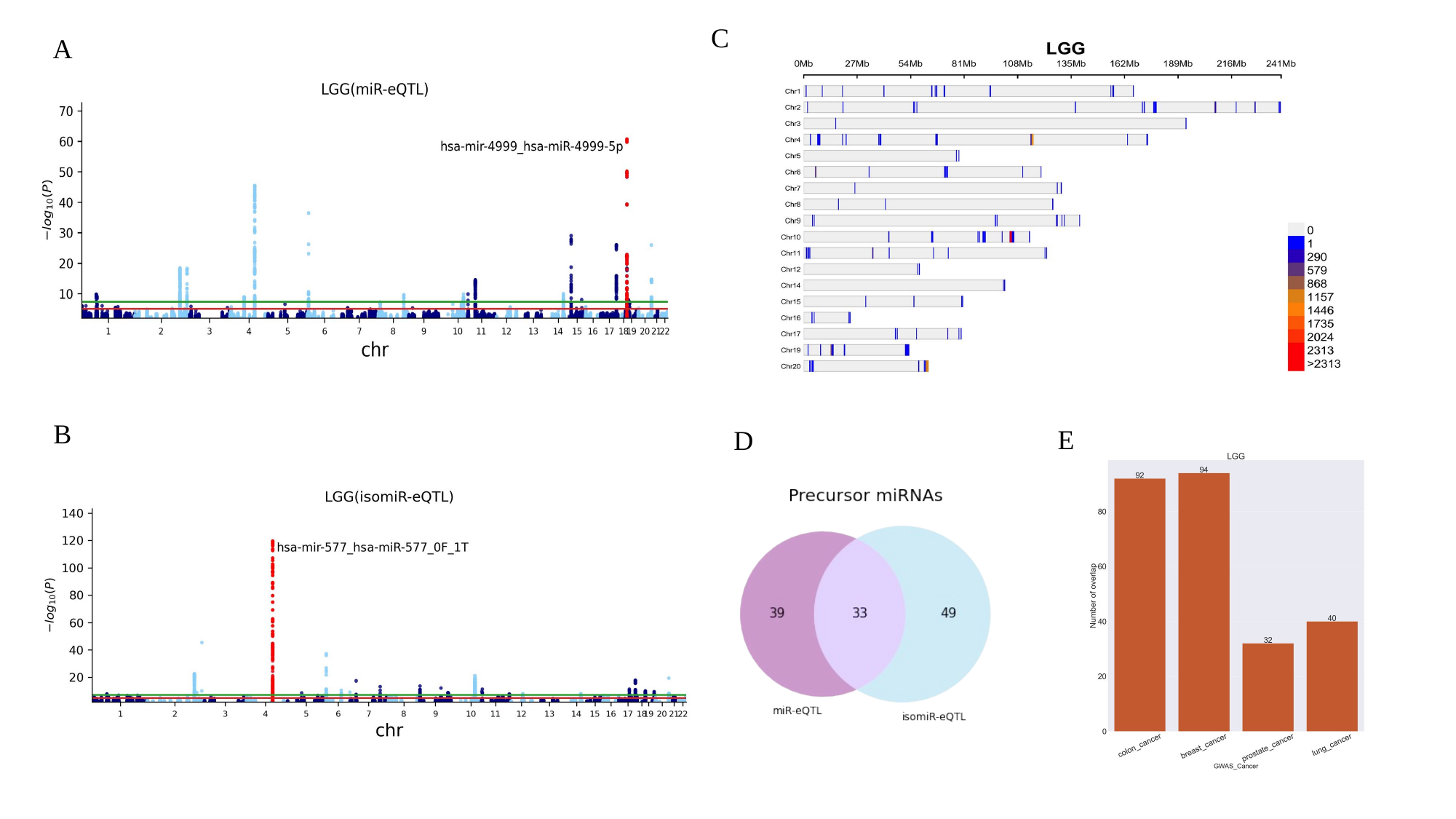

C
A
B
E
D

## Slide 6
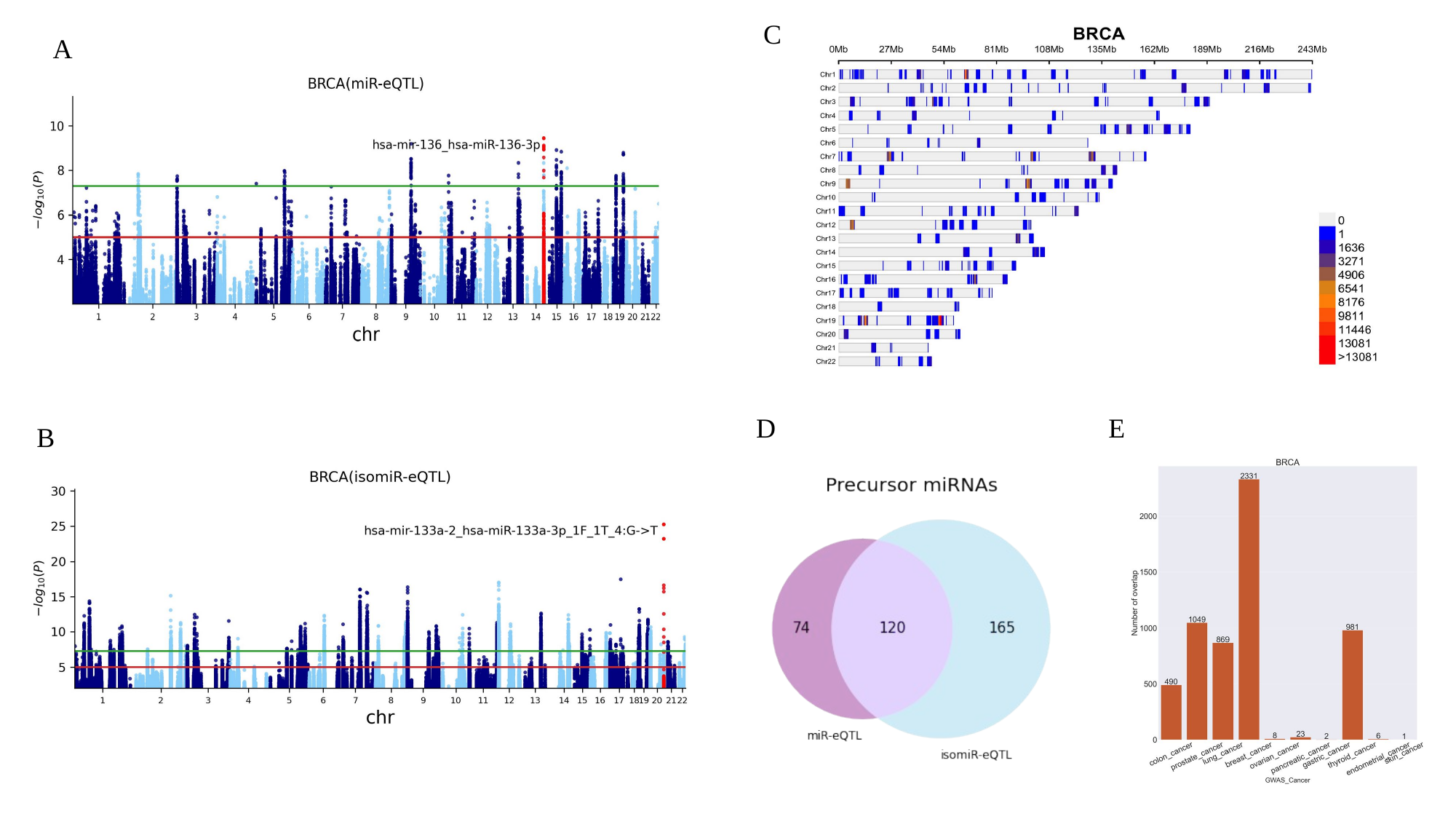

C
A
D
E
B

## Slide 7
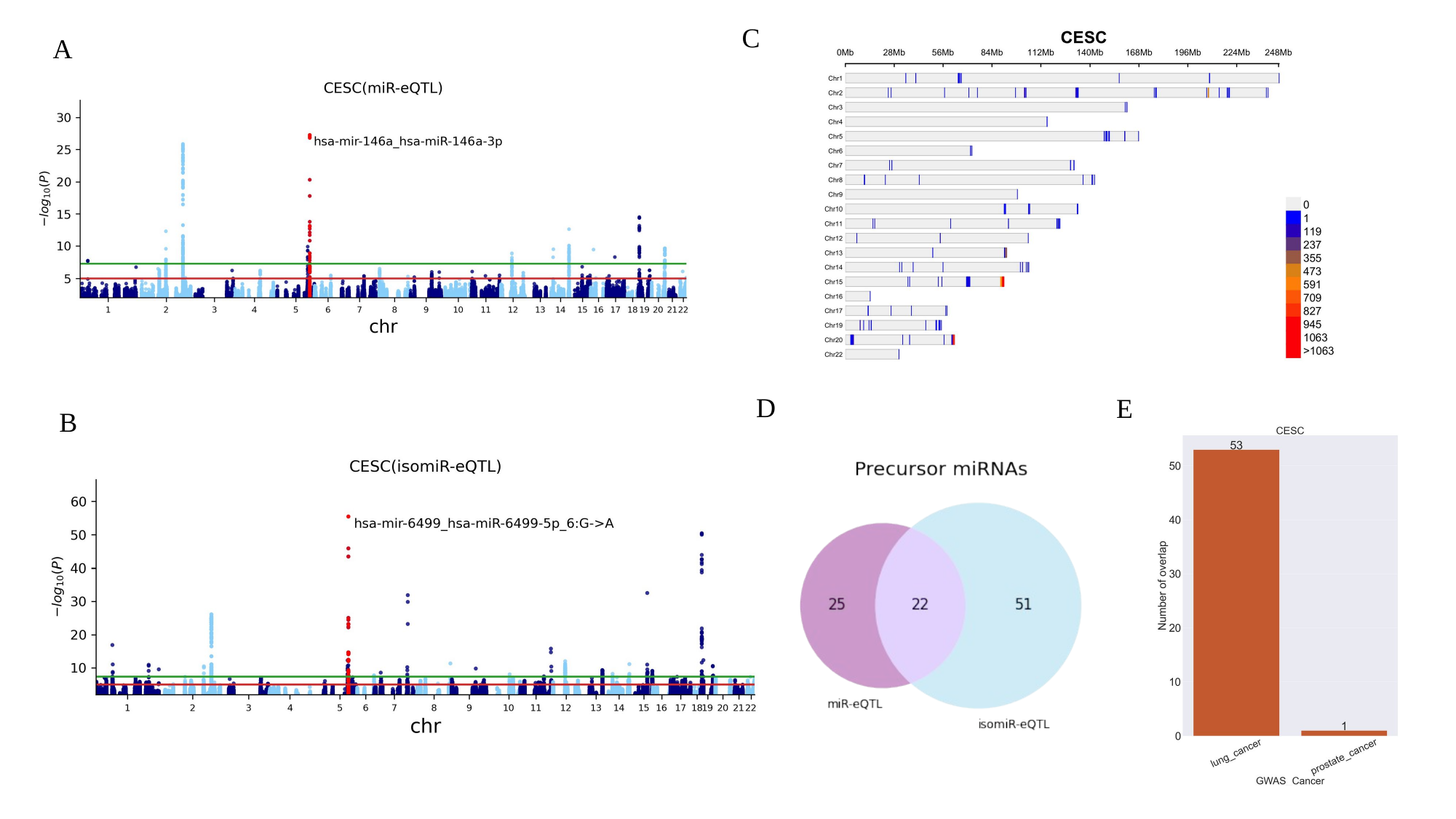

C
A
D
E
B

## Slide 8
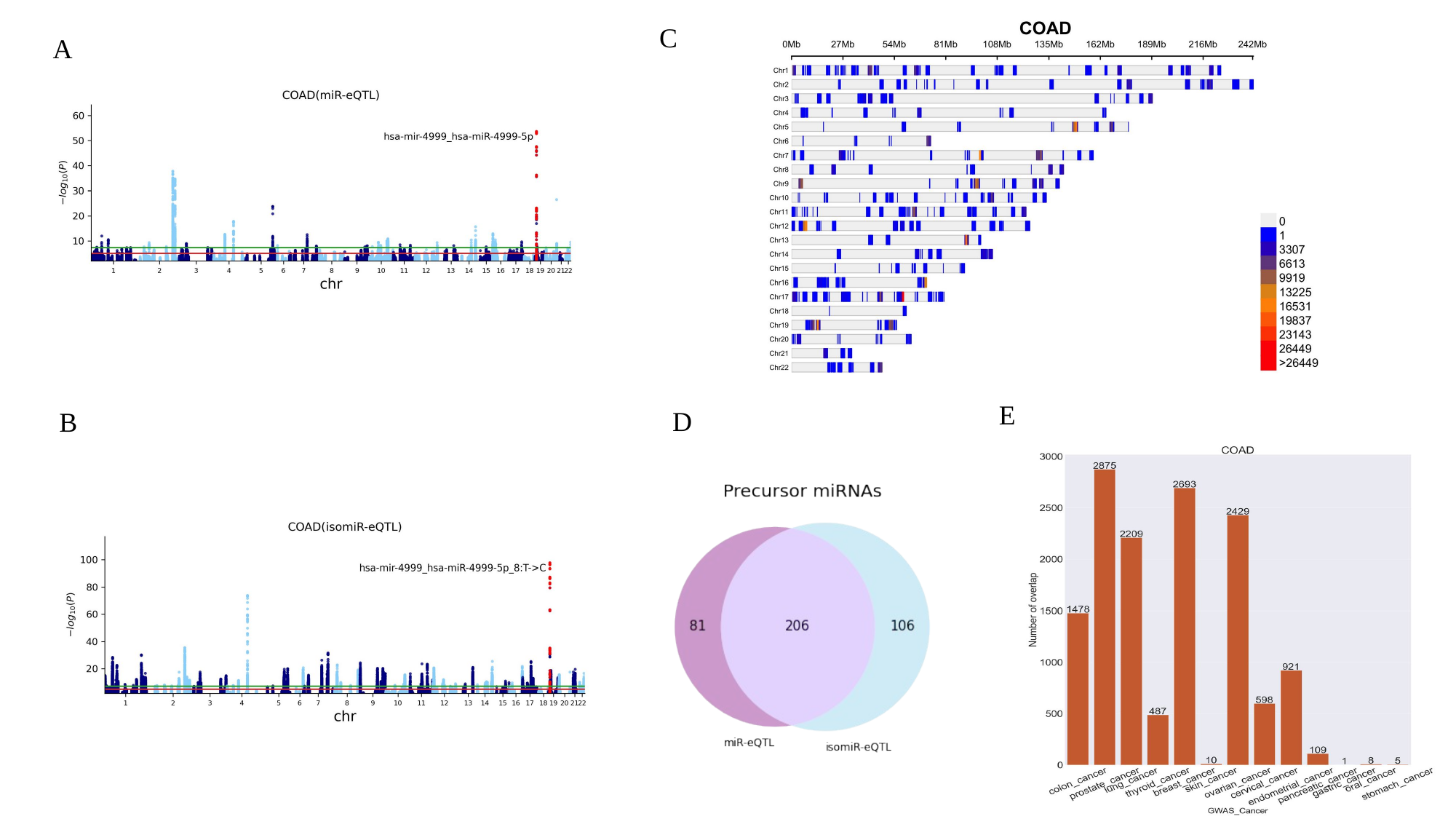

C
A
E
D
B

## Slide 9
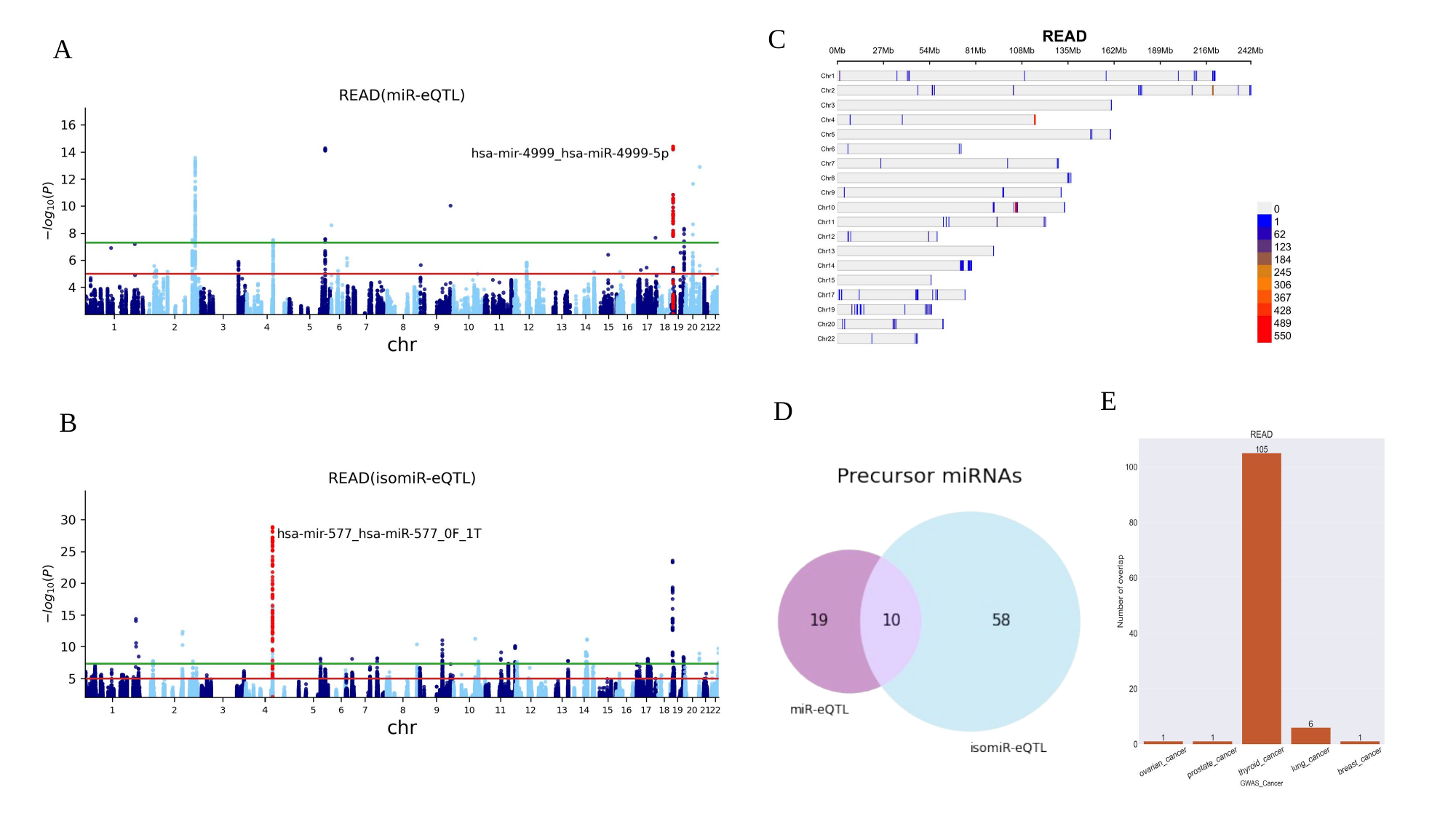

C
A
E
D
B

## Slide 10
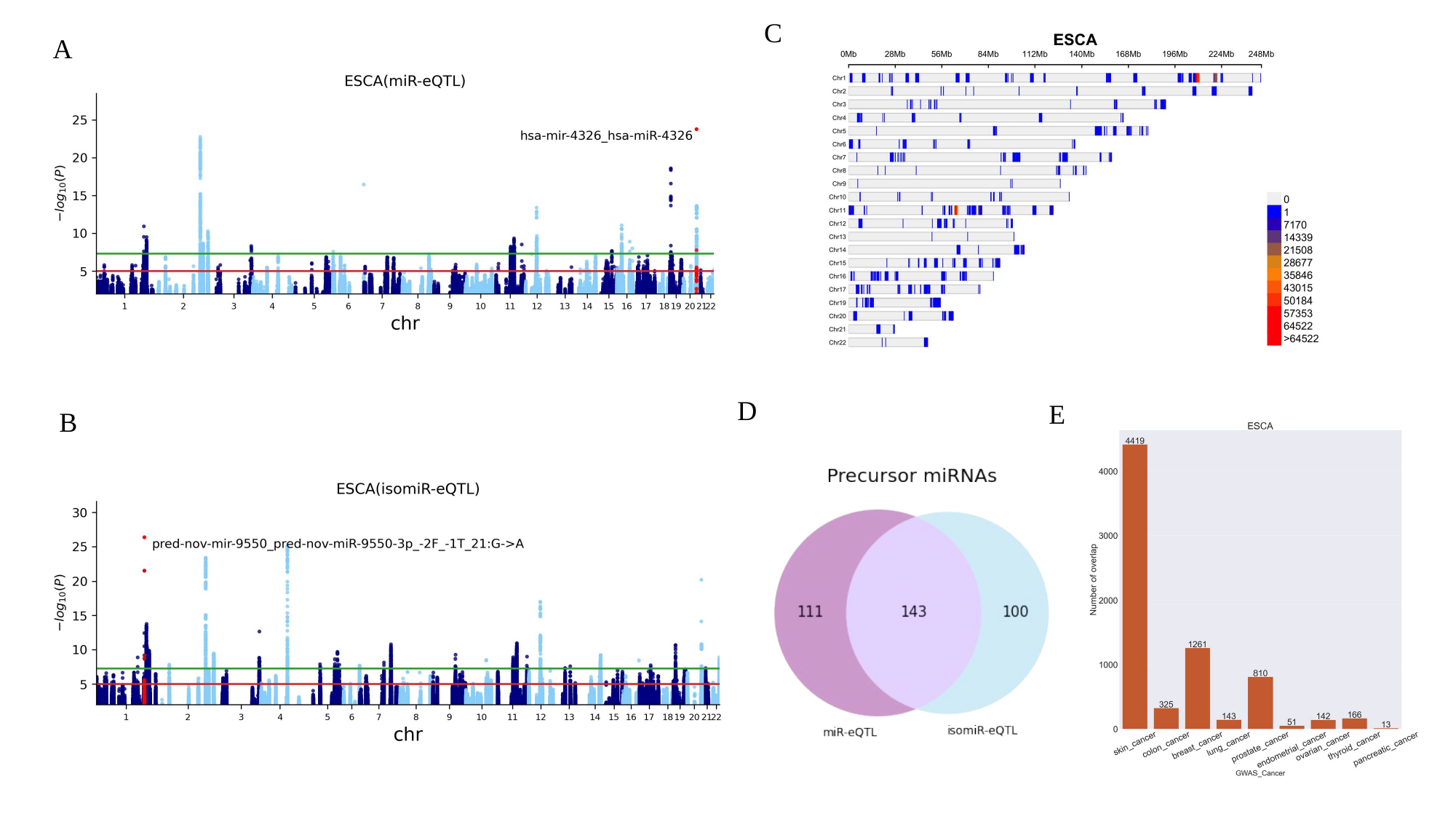

C
A
D
E
B

## Slide 11
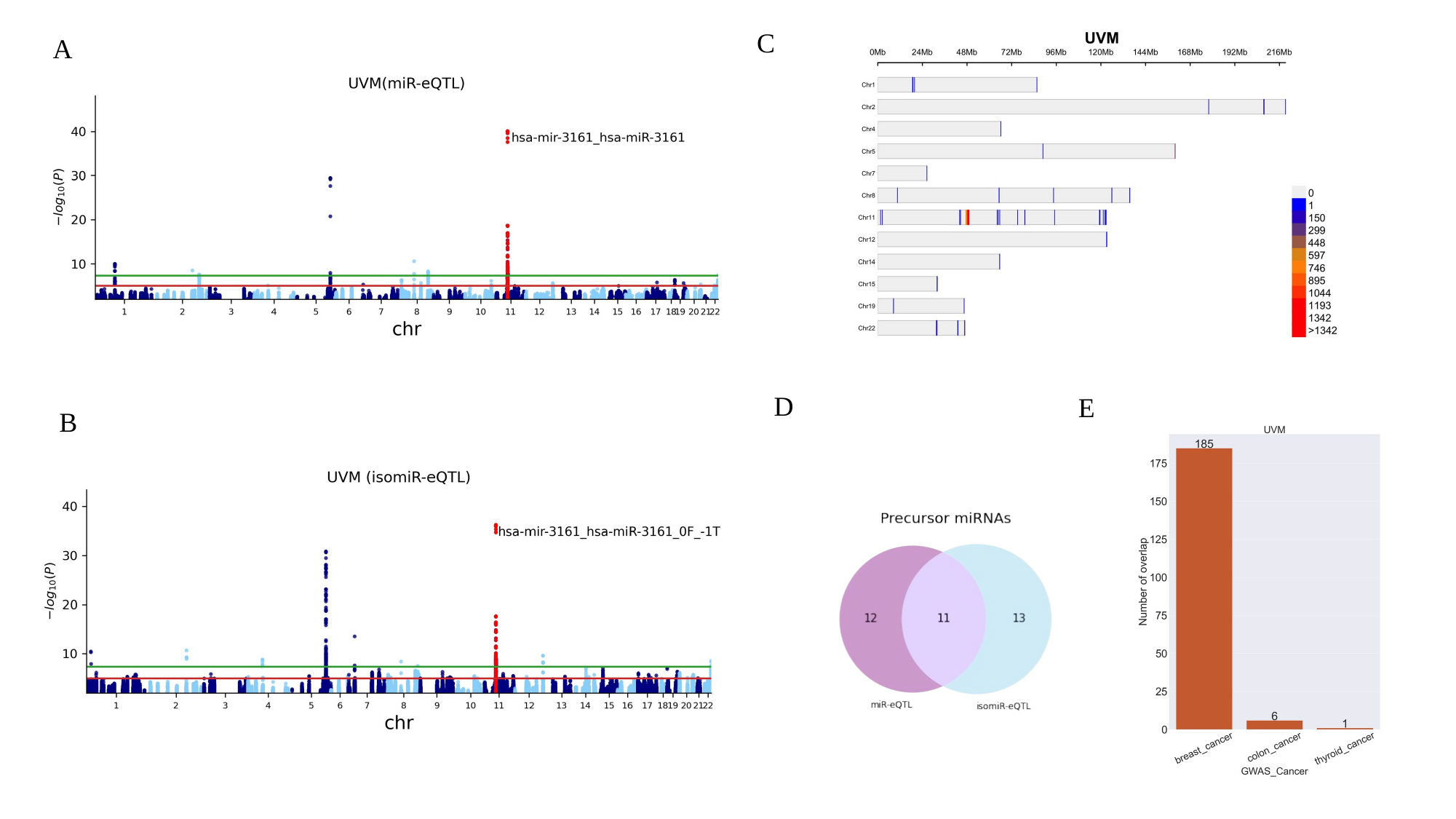

C
A
D
E
B

## Slide 12
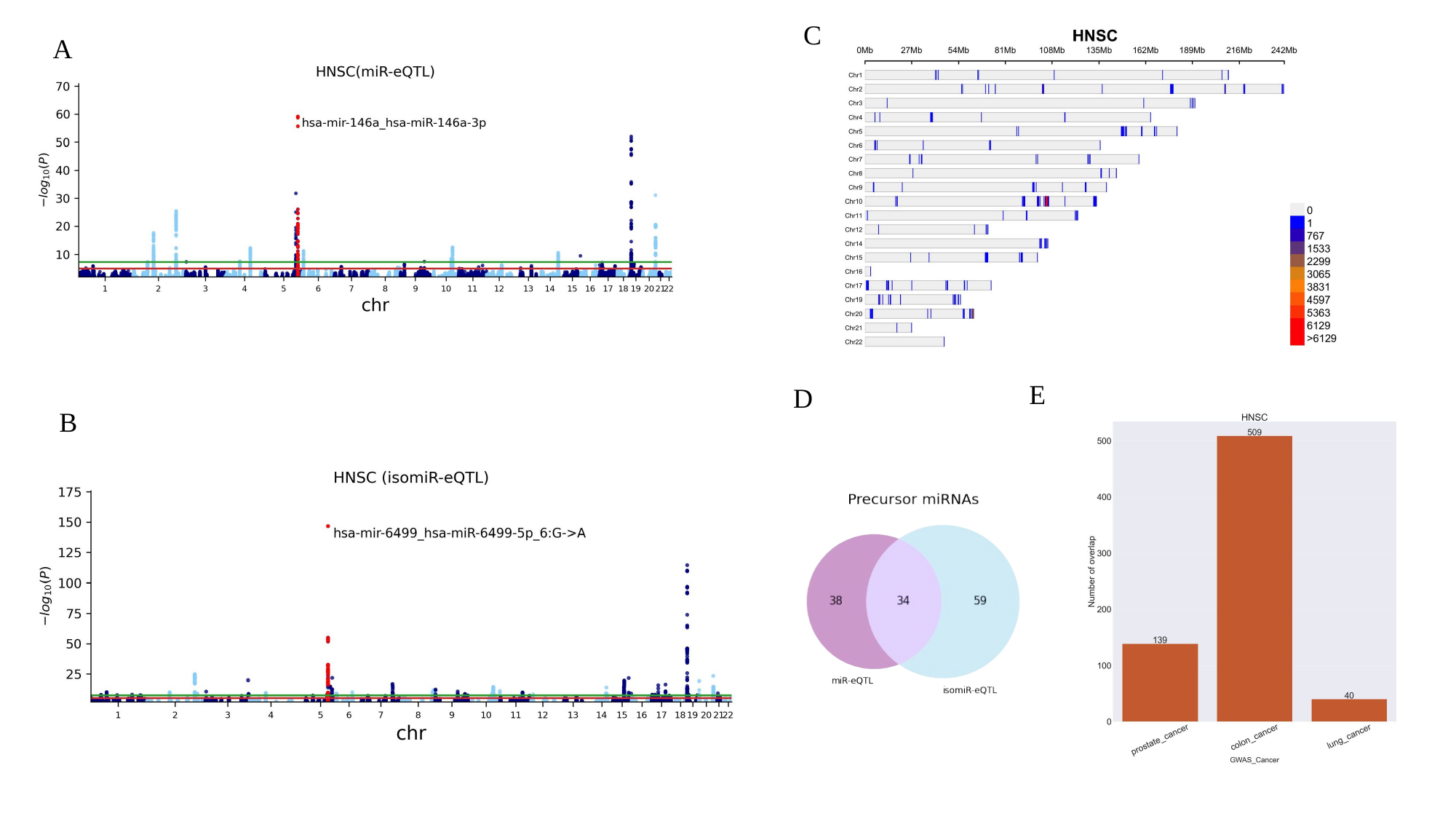

C
A
E
D
B

## Slide 13
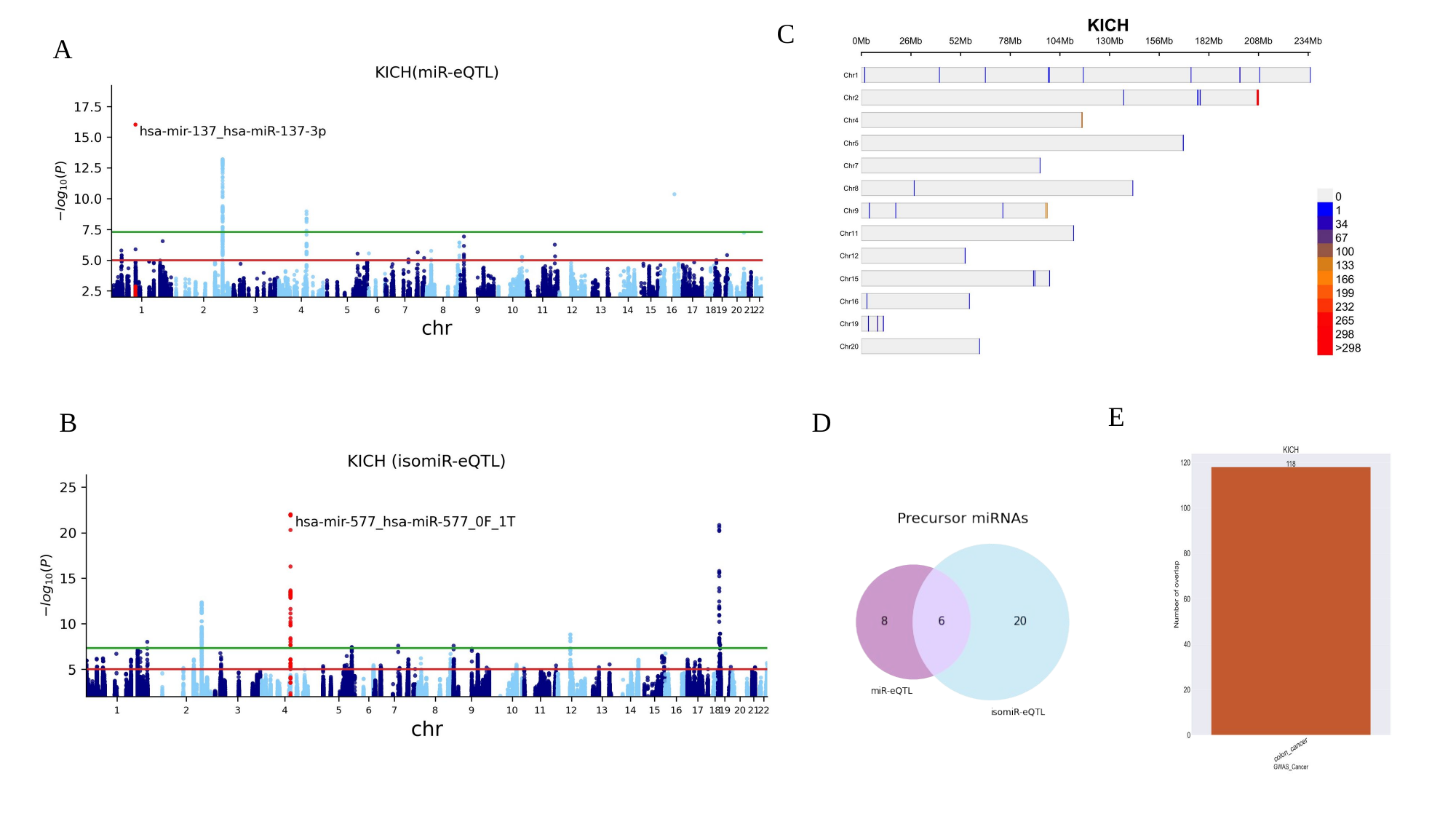

C
A
E
D
B

## Slide 14
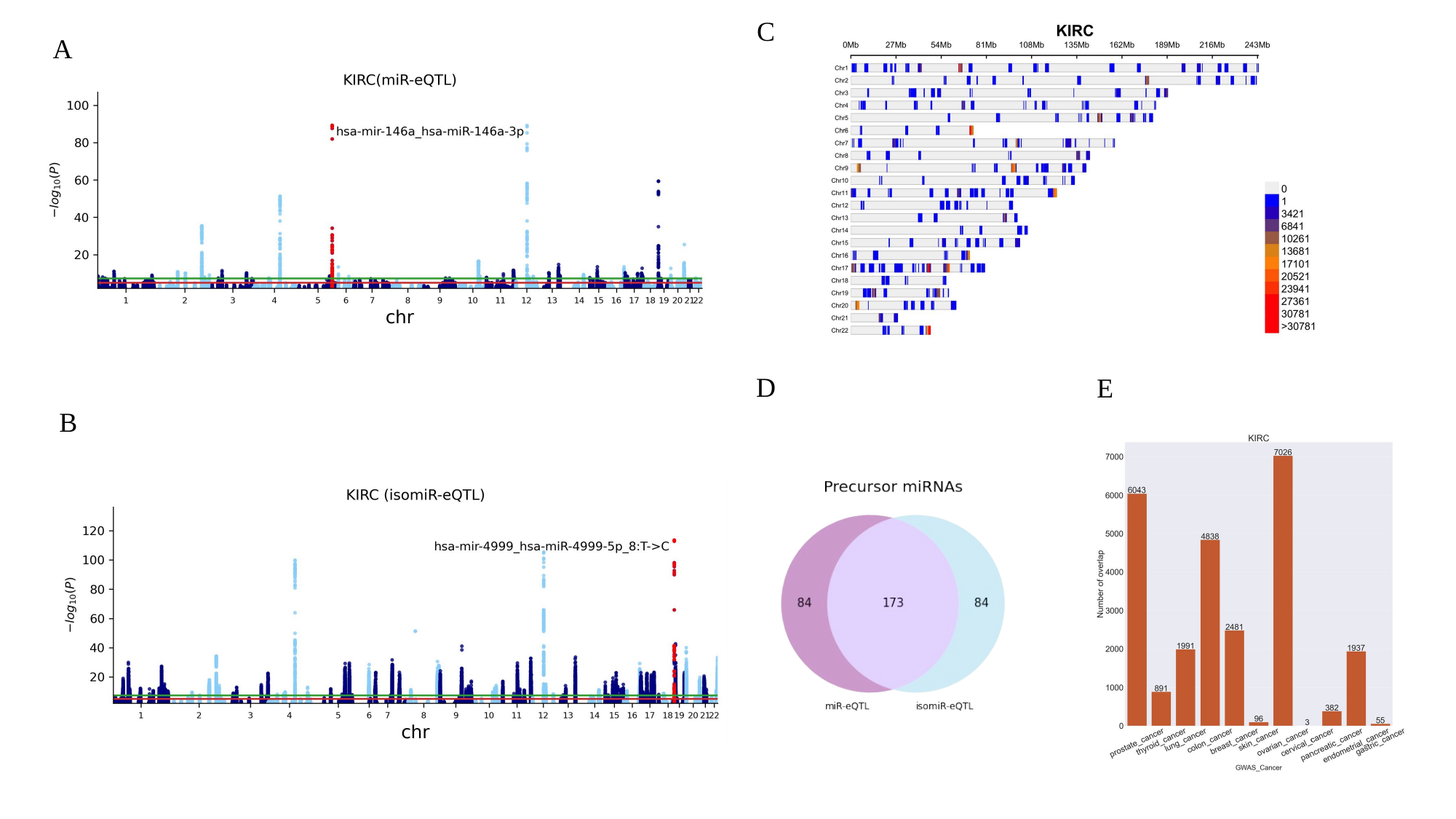

C
A
D
E
B

## Slide 15
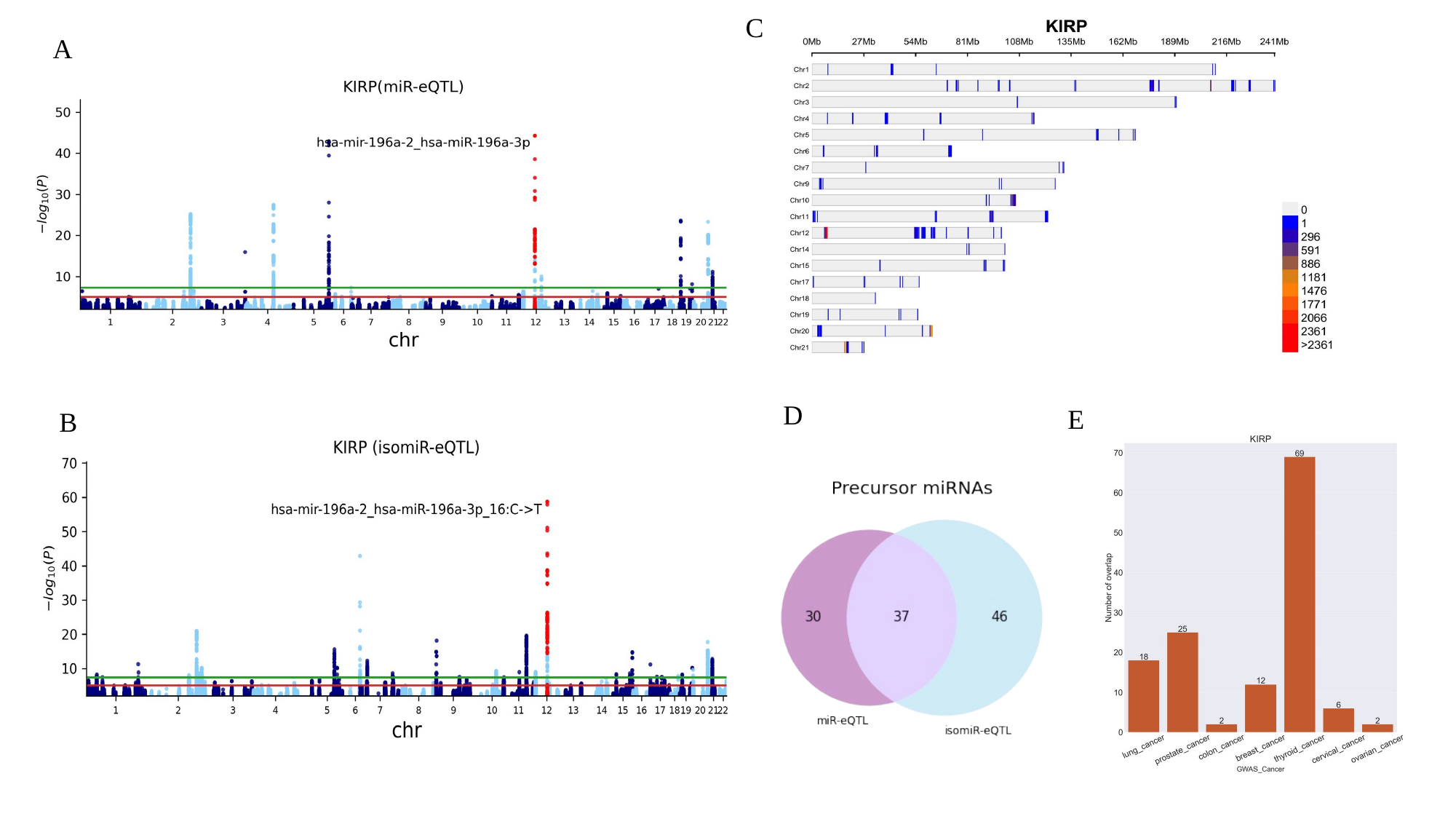

C
A
D
E
B

## Slide 16
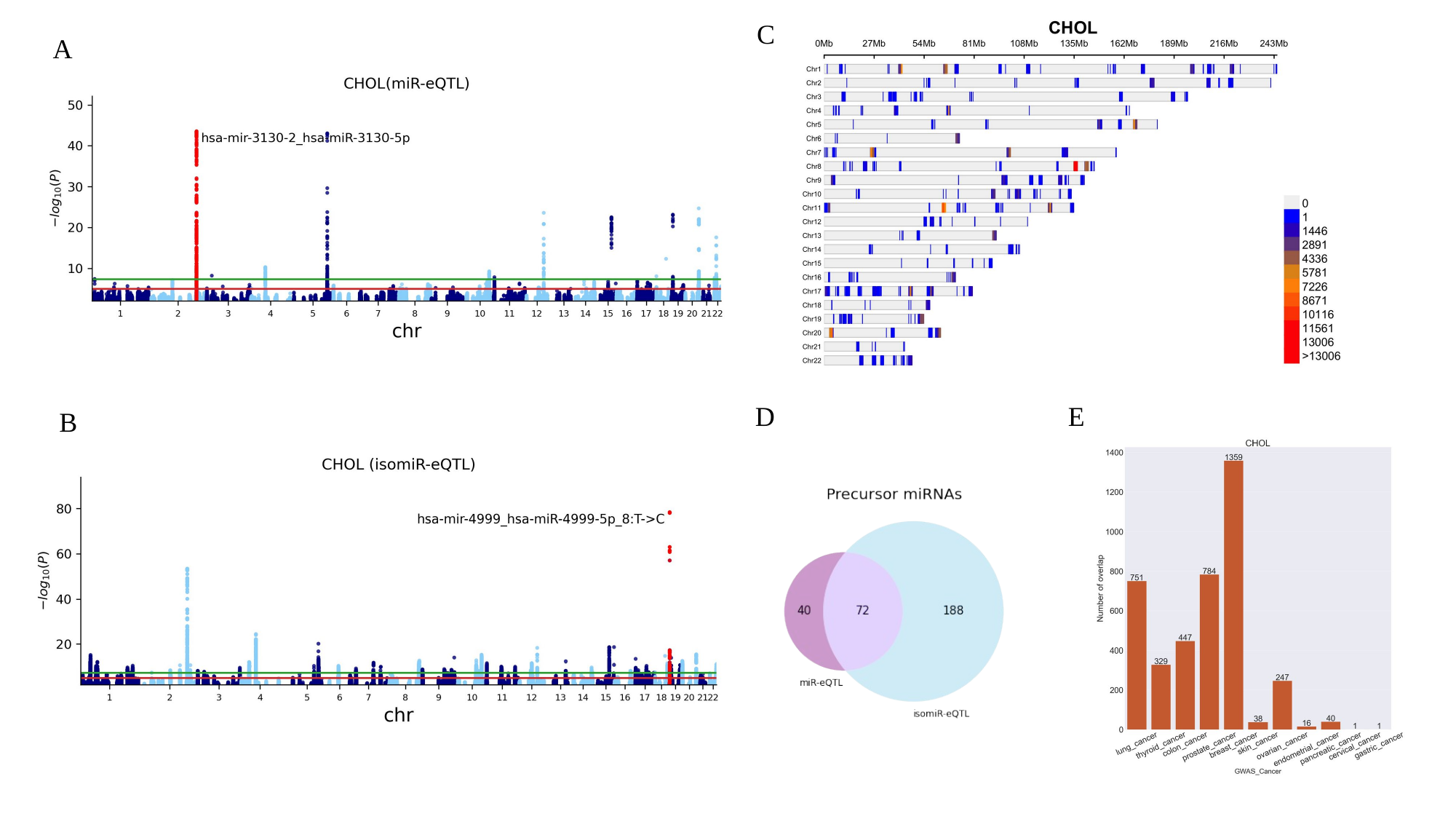

C
A
D
E
B

## Slide 17
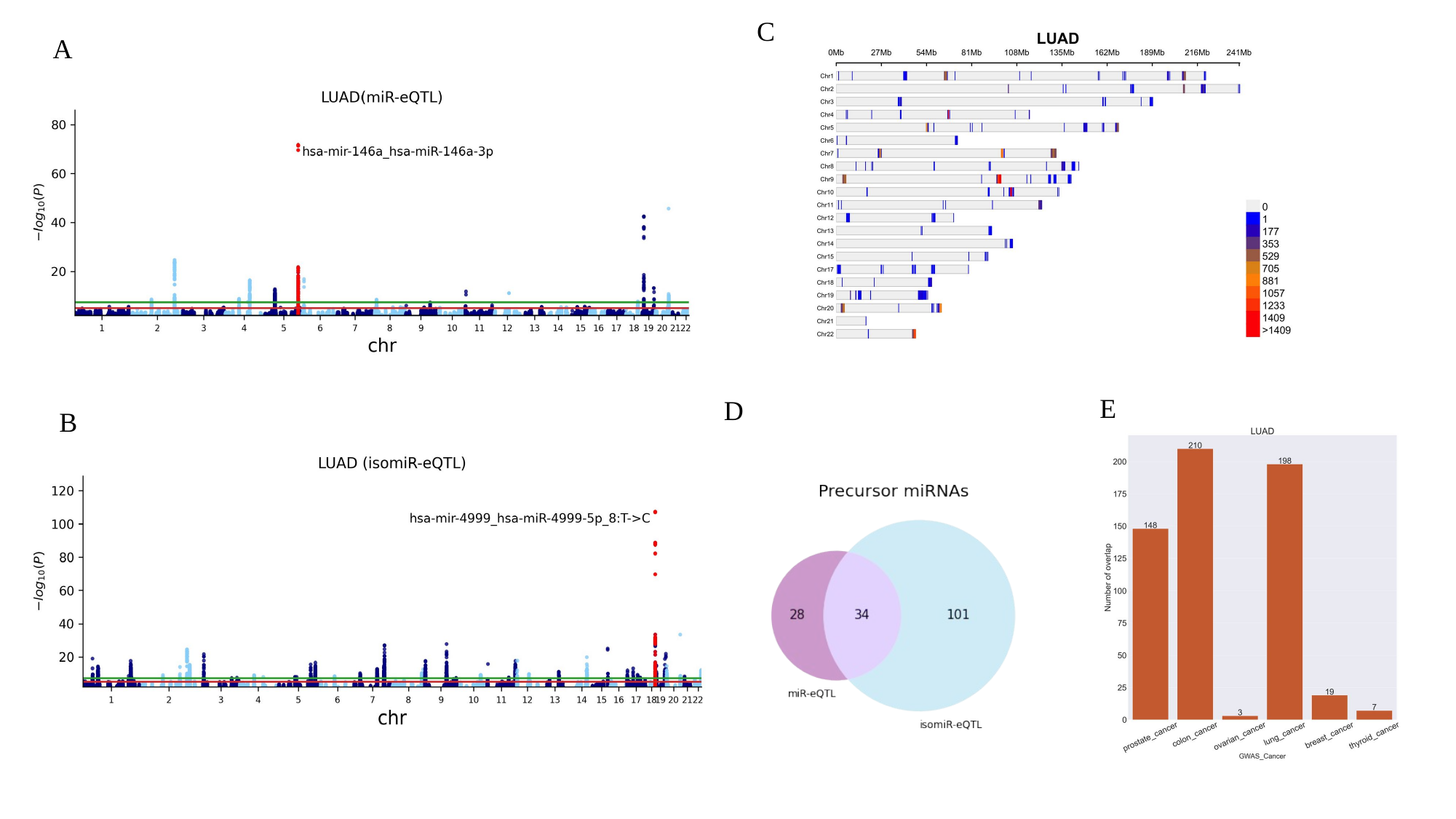

C
A
E
D
B

## Slide 18
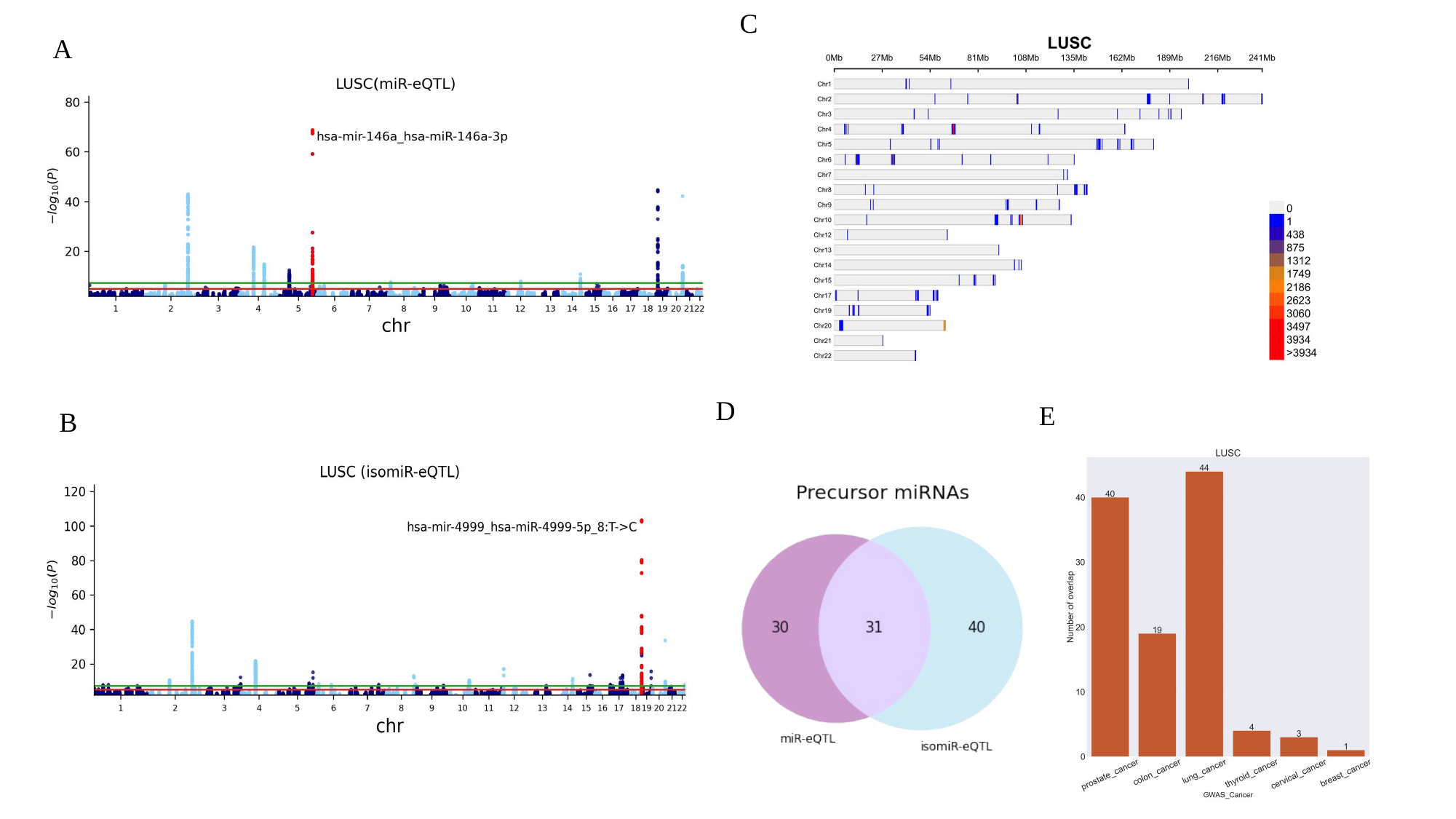

C
A
D
E
B

## Slide 19
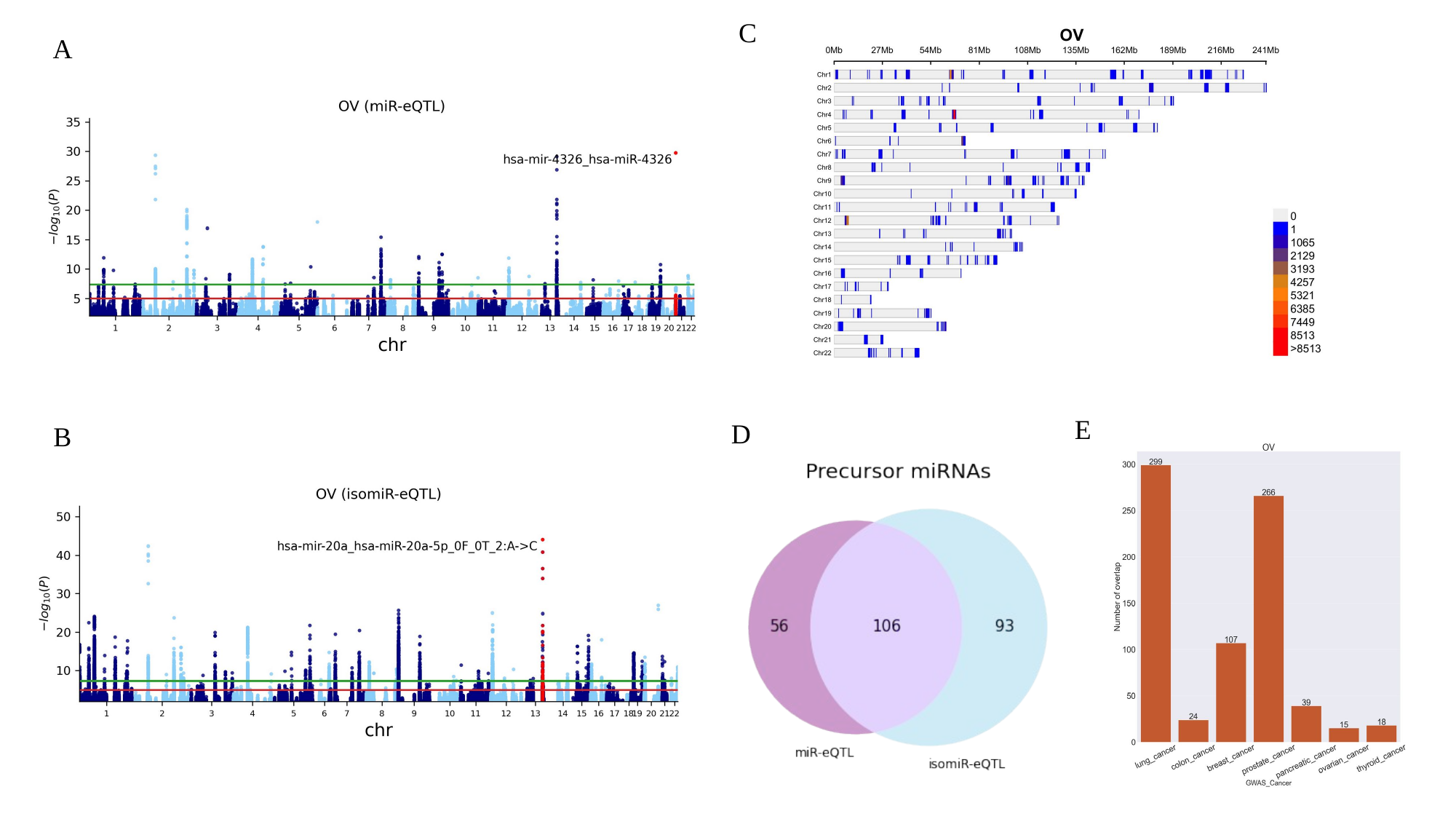

C
A
E
D
B

## Slide 20
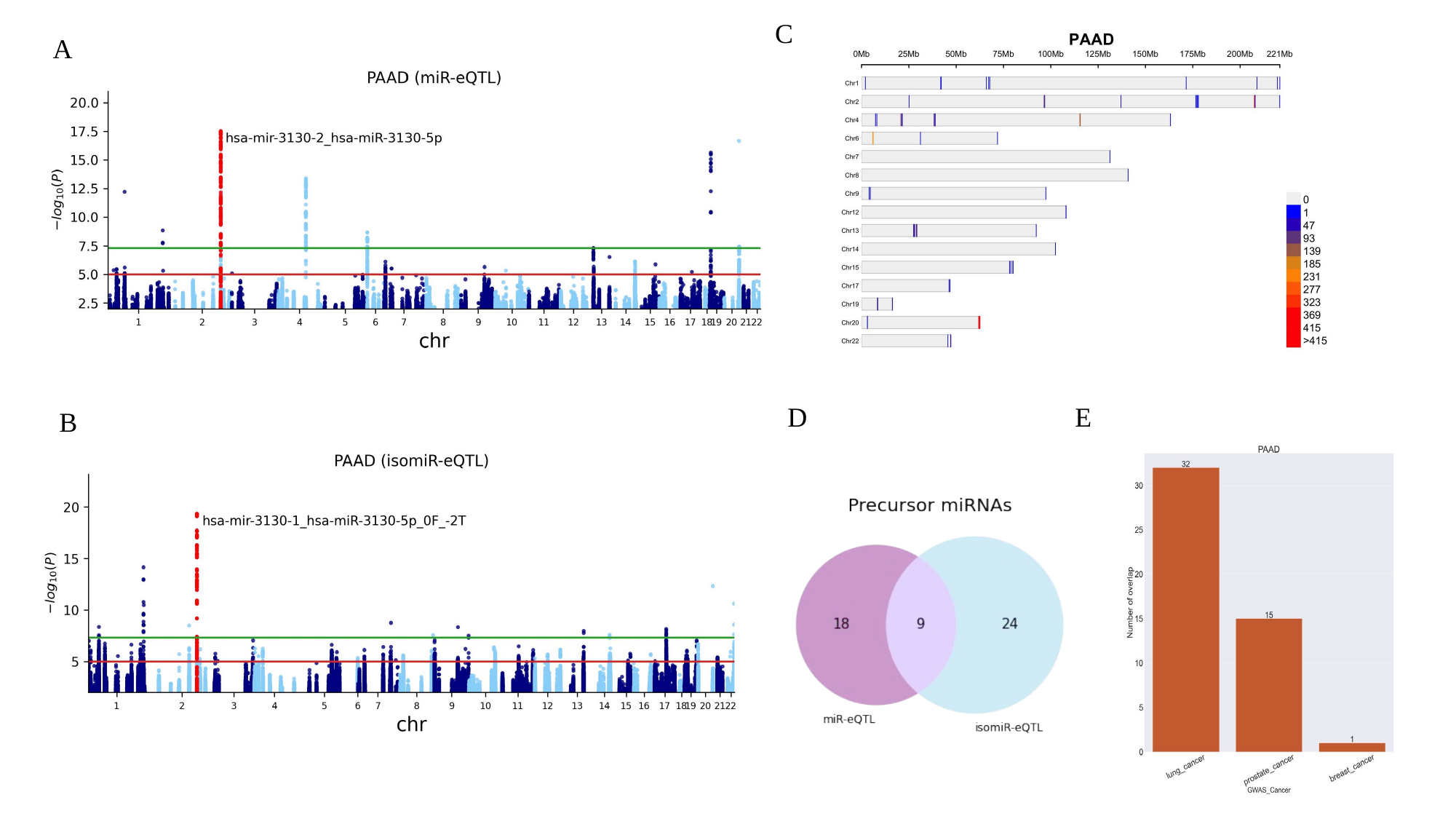

C
A
D
E
B

## Slide 21
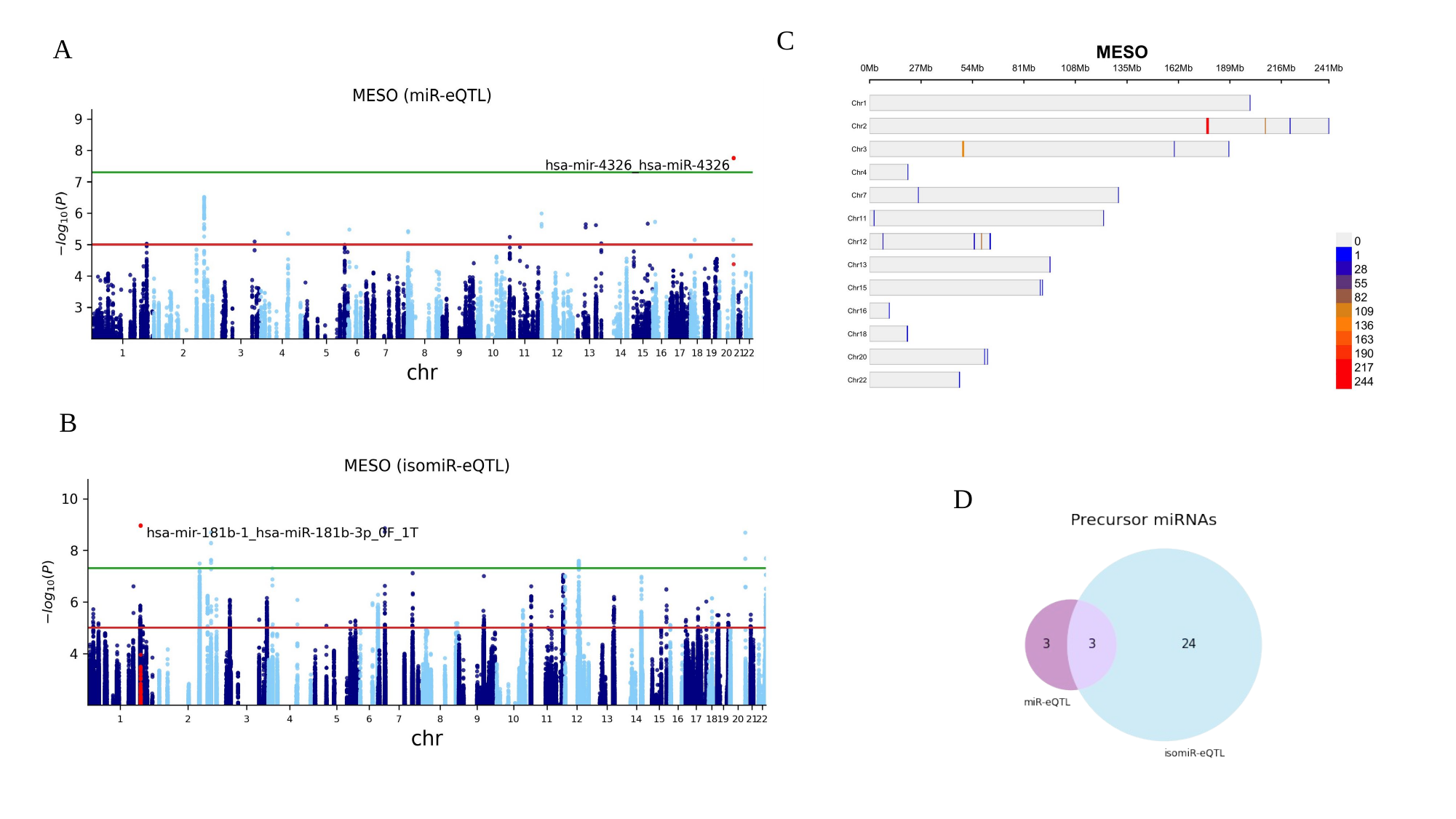

C
A
B
D

## Slide 22
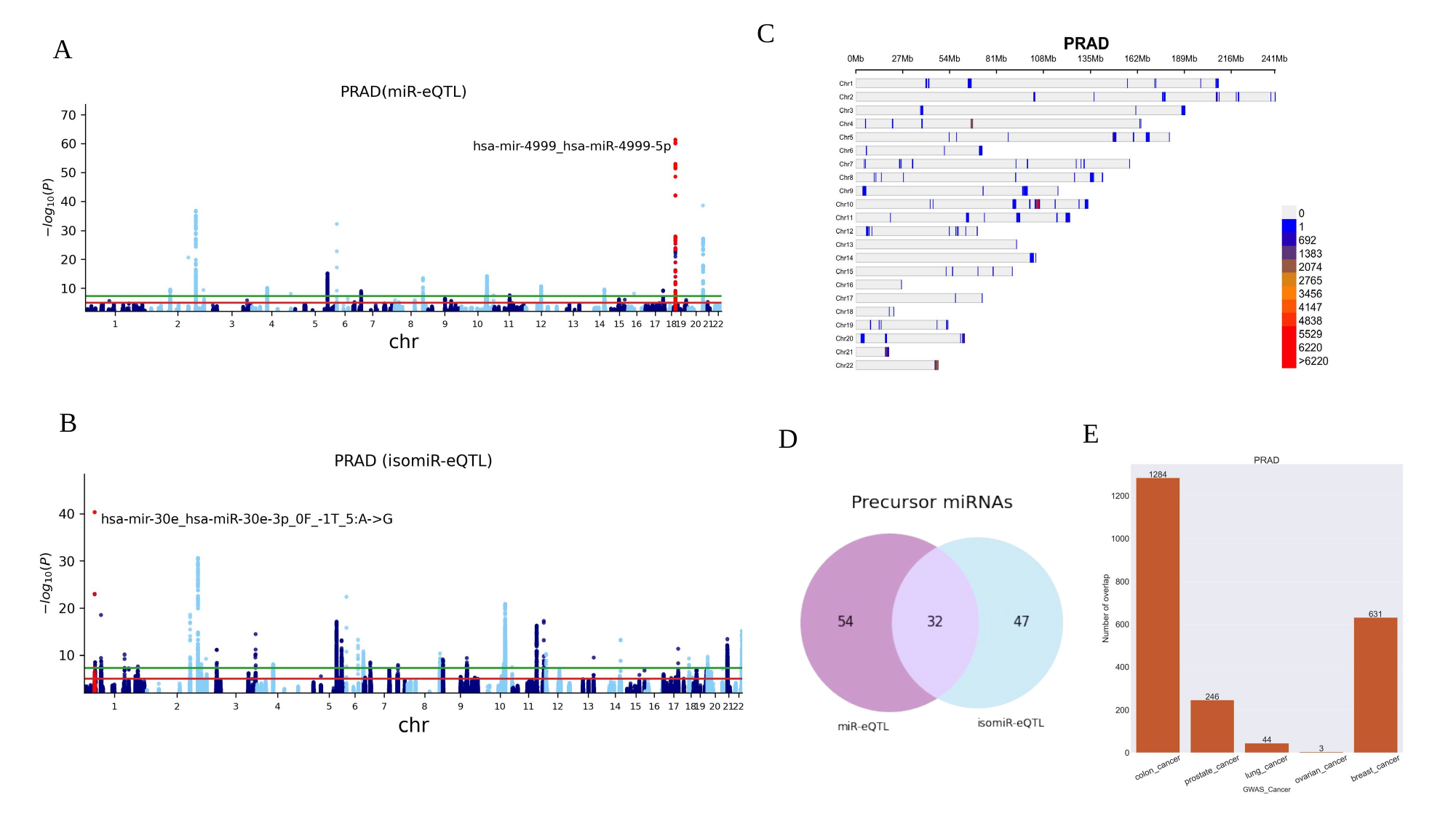

C
A
B
E
D

## Slide 23
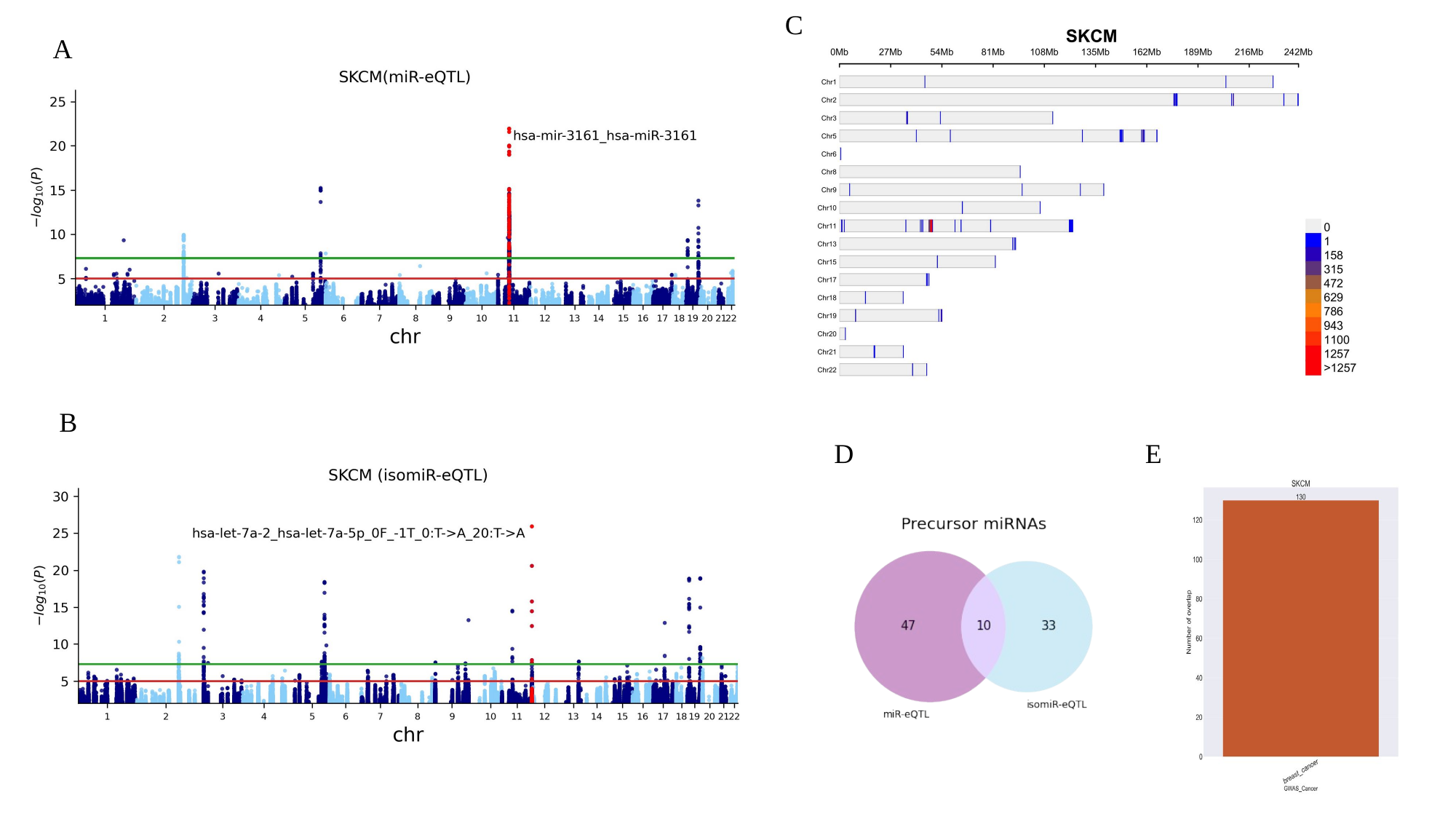

C
A
B
E
D

## Slide 24
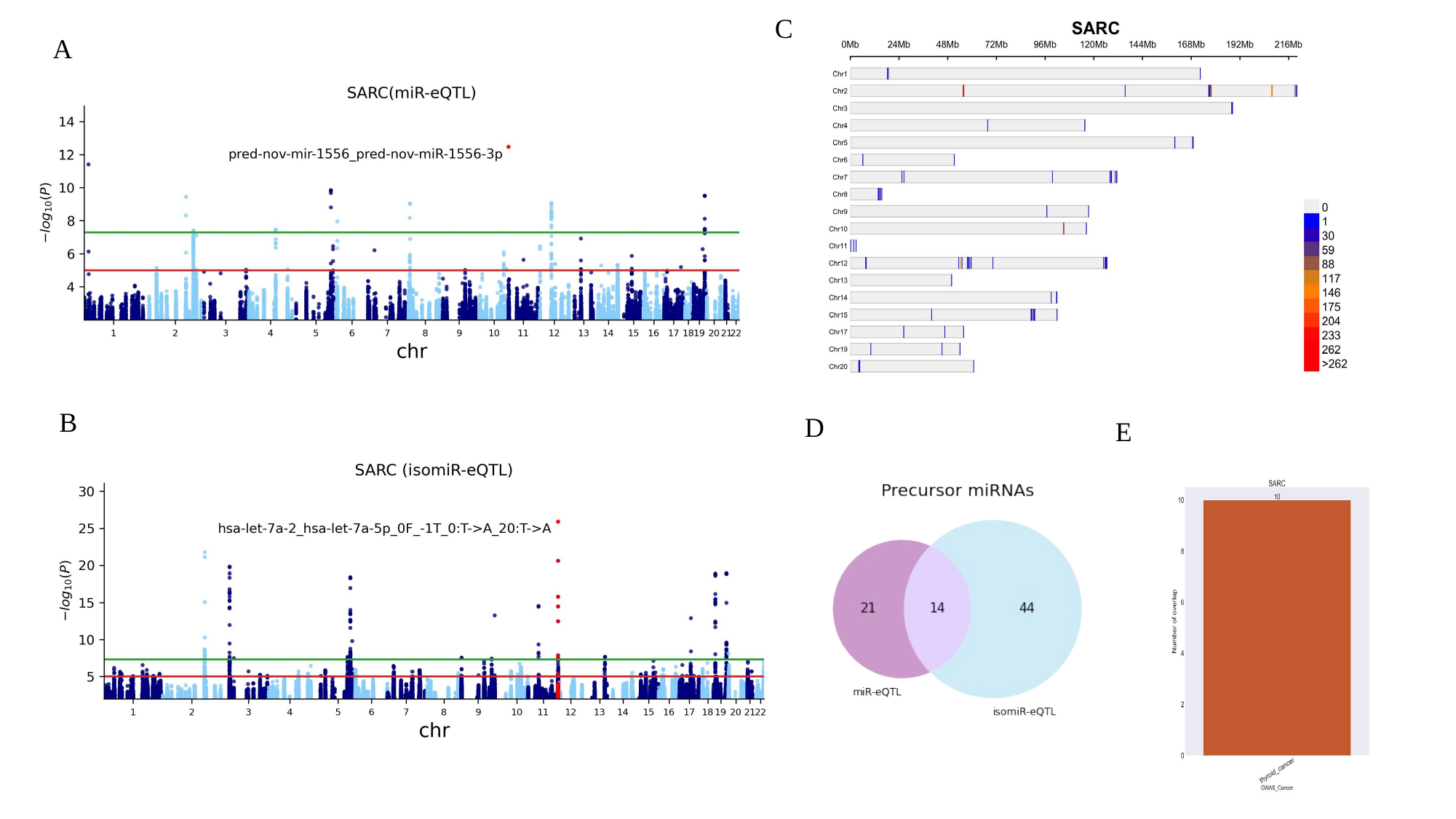

C
A
B
D
E

## Slide 25
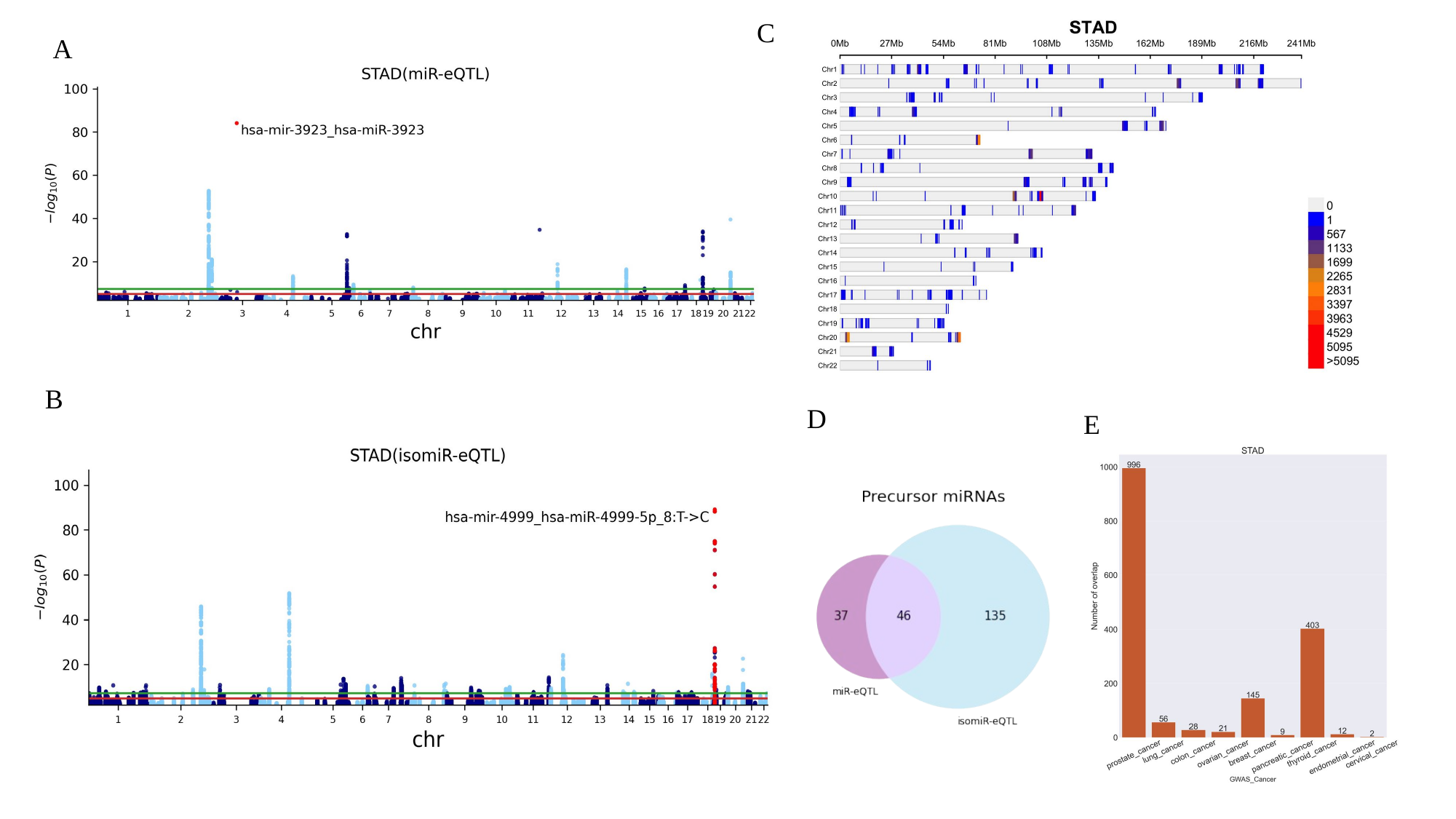

C
A
B
D
E

## Slide 26
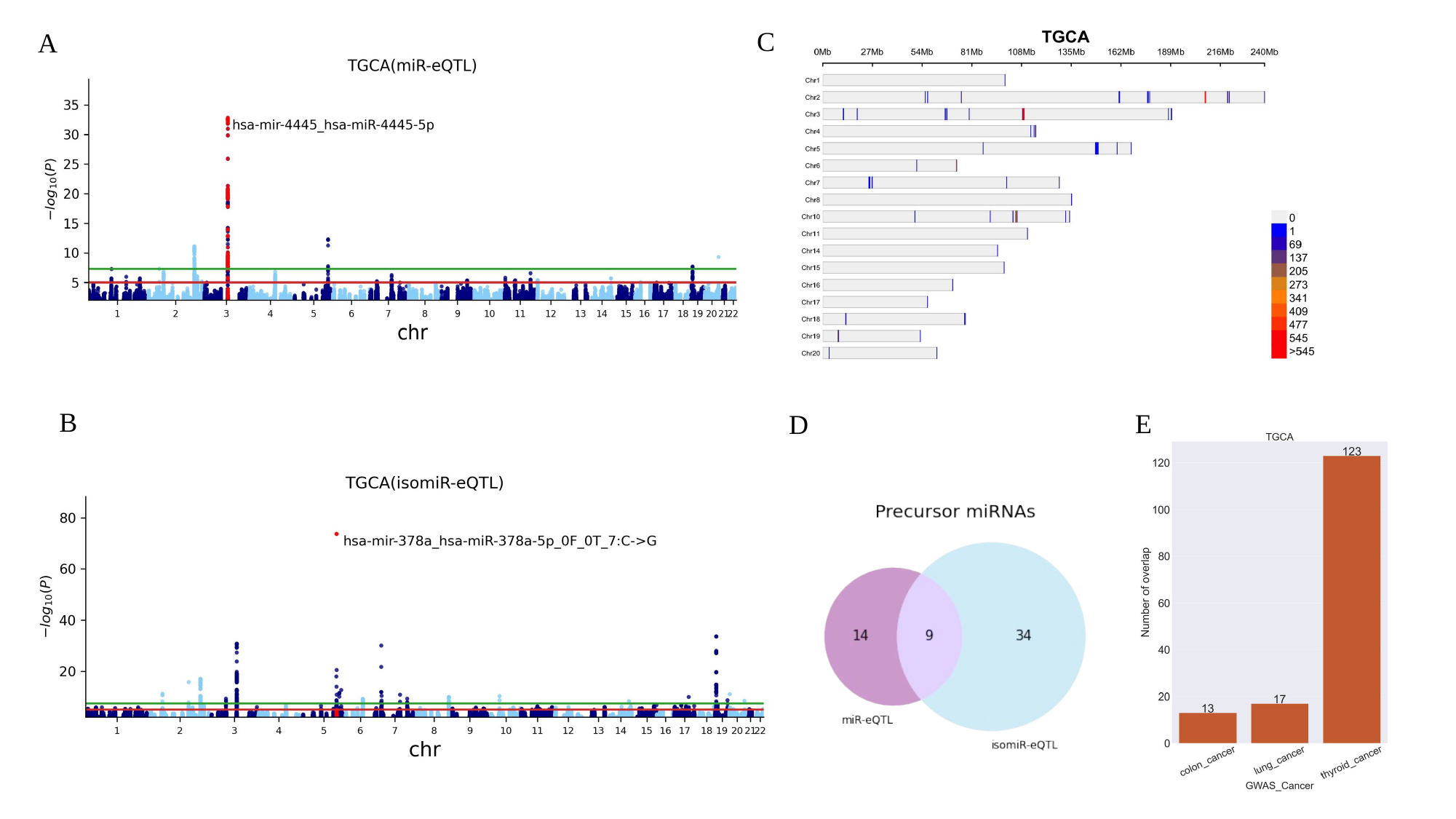

C
A
B
E
D

## Slide 27
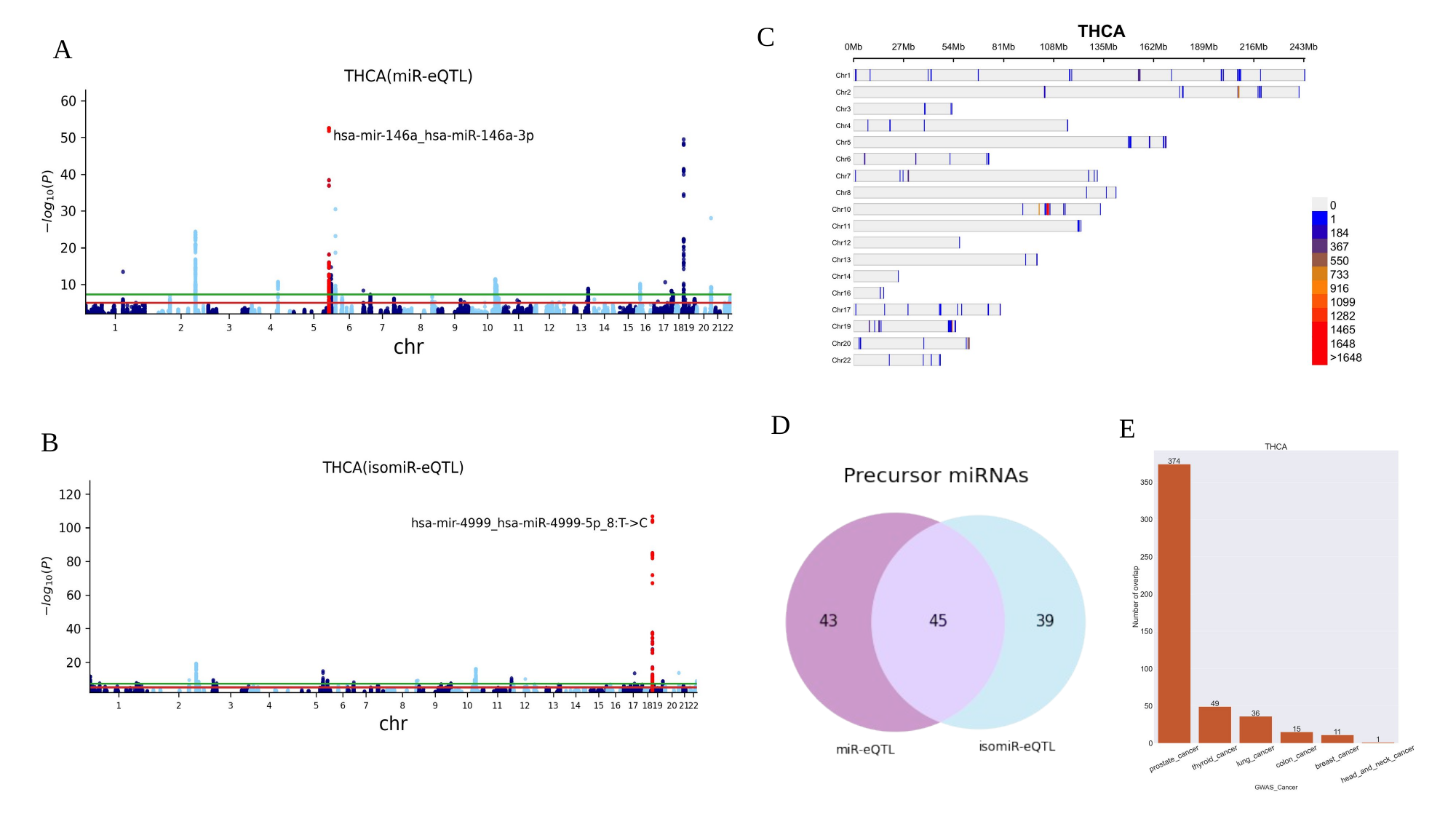

C
A
D
E
B

## Slide 28
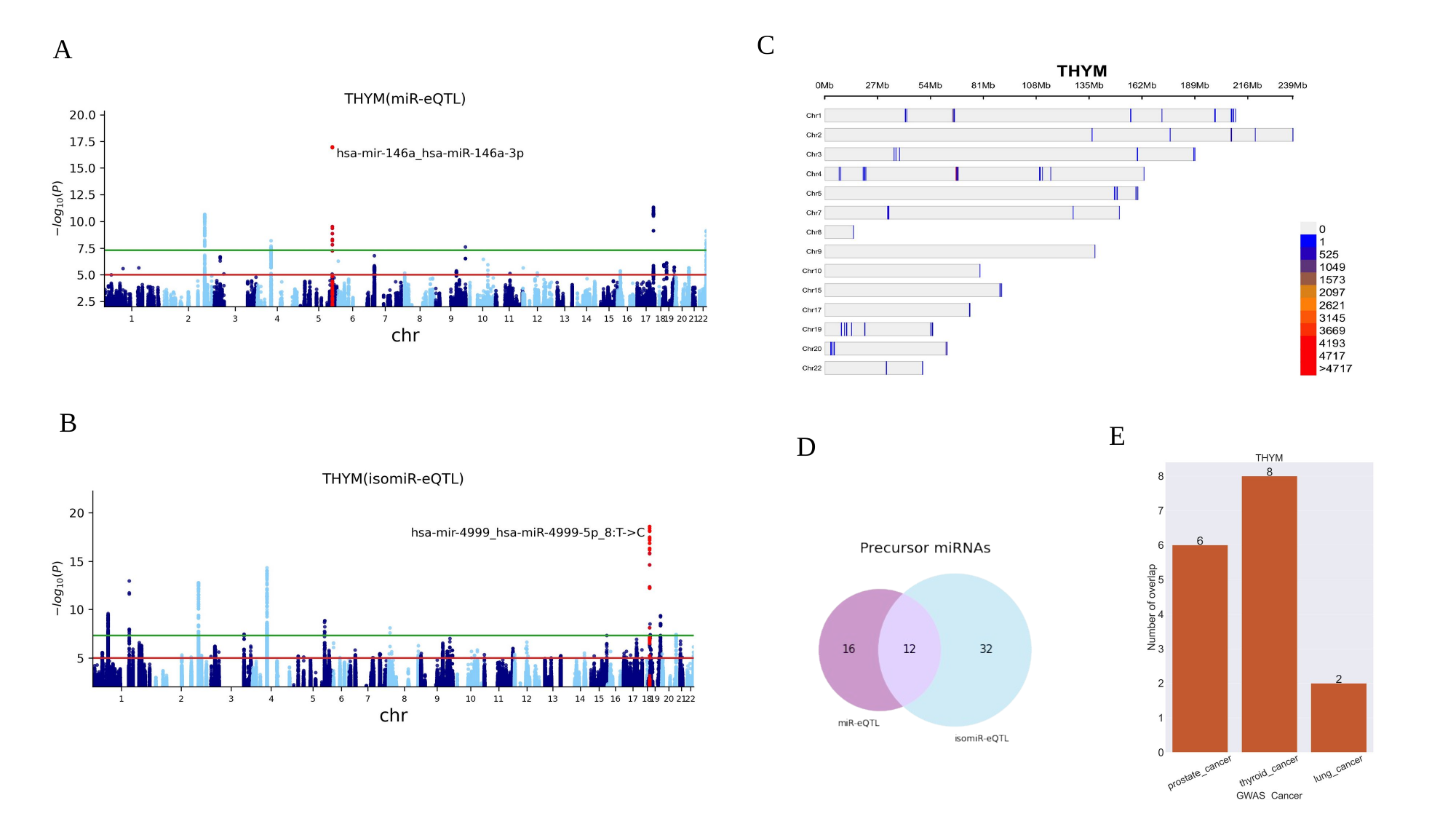

C
A
B
E
D

## Slide 29
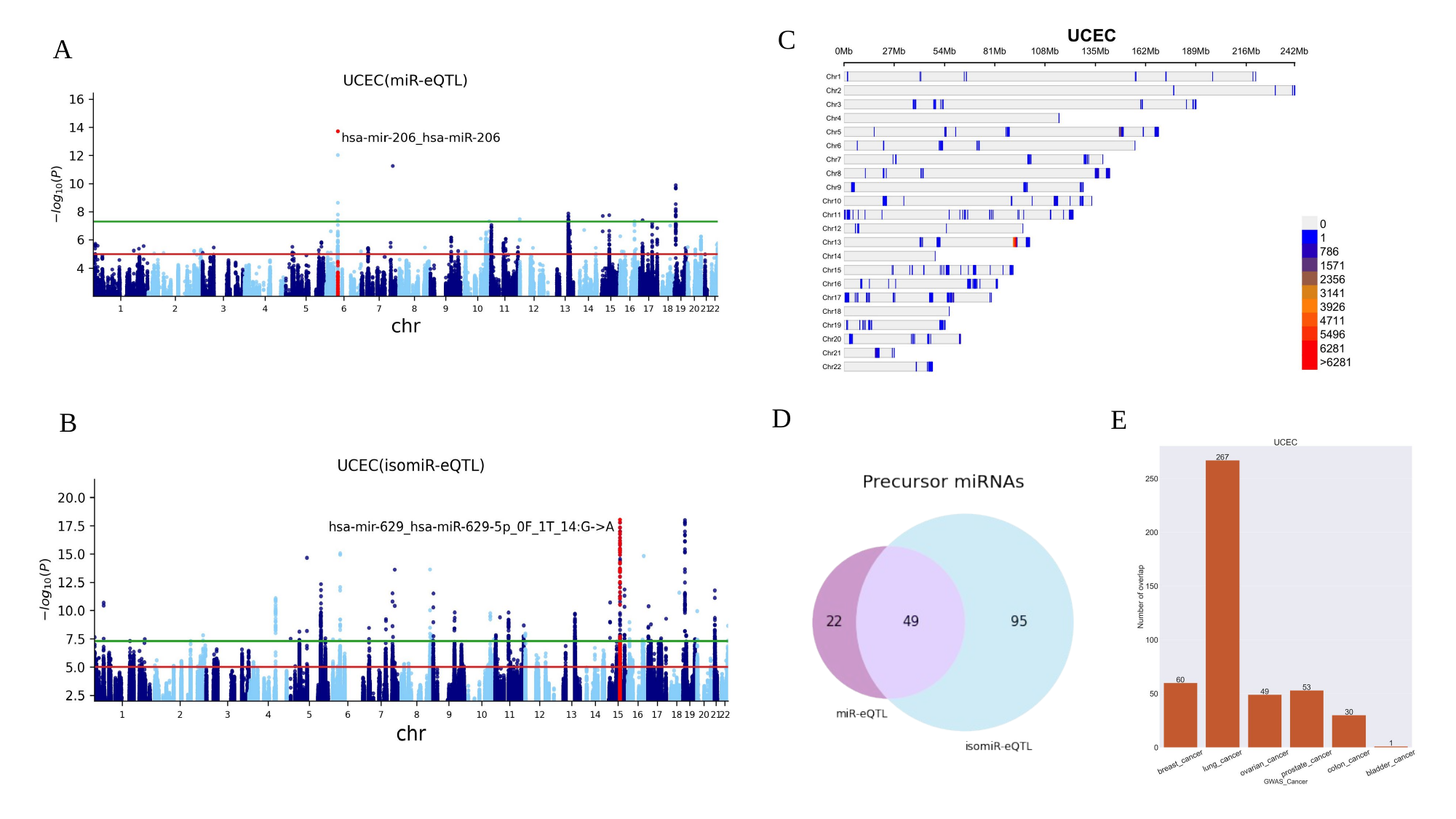

C
A
D
E
B

## Slide 30
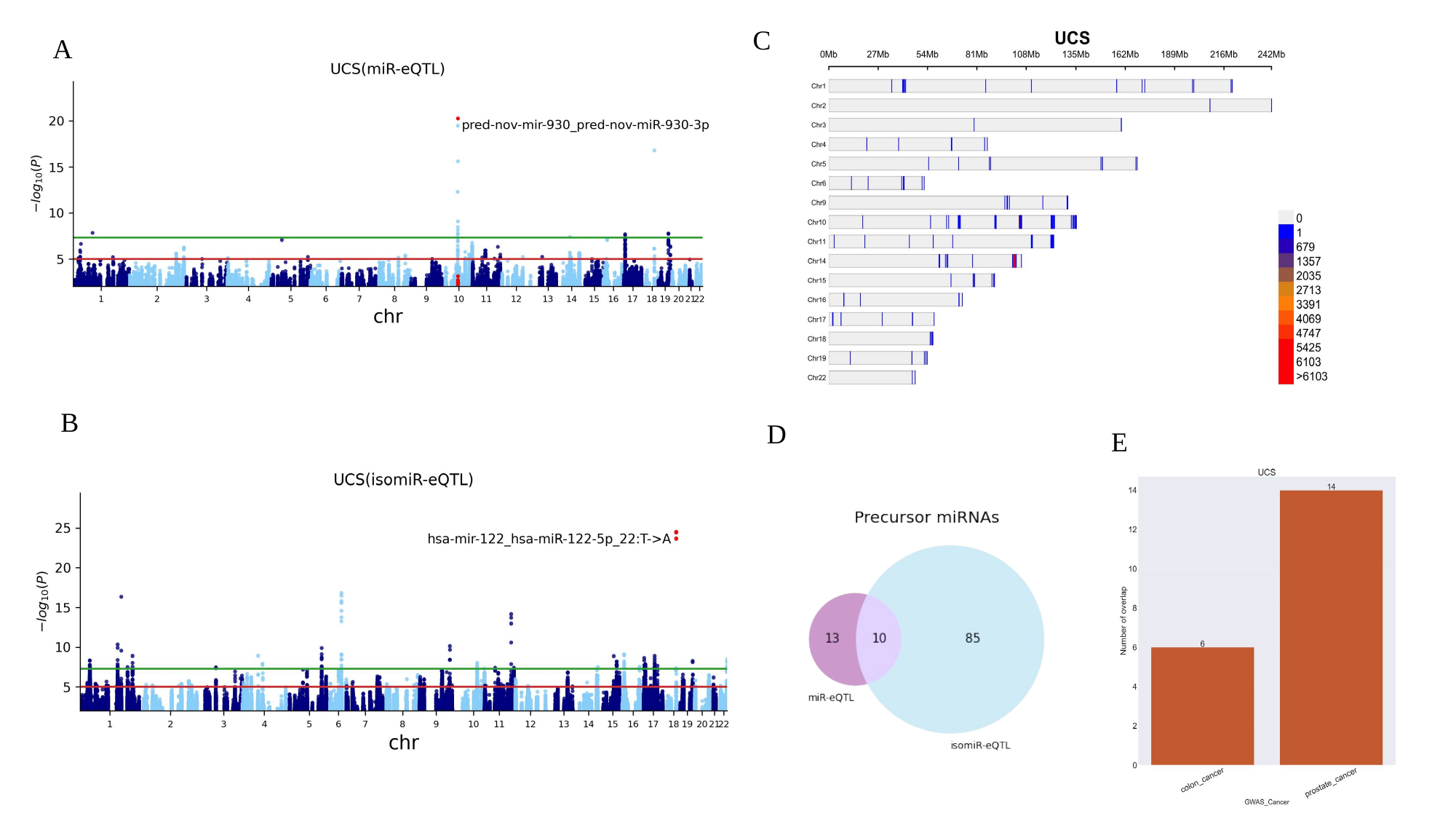

C
A
B
D
E
